# Epidemiology and determinants of dyslipidemia in Iranian population: Results from the PERSIAN cohort

**DOI:** 10.1371/journal.pone.0352516

**Published:** 2026-07-17

**Authors:** Nazgol Motamed-Gorji, Sadaf G. Sepanlou, Marjan Moallemian Isfahani, Ali Ahmadi, Meysam Alipour, Azizallah Dehghan, Pedram Ebrahimnejad, Mohhamad Reza Fattahi, Masoumeh Goddusi Johari, Zahra Jamali, Mahmoud Ali Kaykhaei, Seyyedadel Khoshbou_Garebagh, Fariborz Mansour-Ghanaei, Somaieh Matin, Masoud Mirzaei, Yousef Moradi, Azim Nejatizadeh, Alireza Ostadrahimi, Ayoob Rastegar, Moslem Sedaghattalab, Ebrahim Shakiba, Sareh Eghtesad, Zahra Mohammadi, Hossein Poustchi, Reza Malekzadeh

**Affiliations:** 1 Liver and Pancreatobiliary Diseases Research Center, Digestive Diseases Research Institute, Tehran University of Medical Sciences, Tehran, Iran; 2 Digestive Disease Research Center, Digestive Diseases Research Institute, Tehran University of Medical Sciences, Tehran, Iran; 3 Modeling in Health Research Center, Shahrekord University of Medical Sciences, Shahrekord, Iran; 4 Department of Nutrition, Shoushtar Faculty of Medical Sciences, Shoushtar, Iran; 5 Noncommunicable Diseases Research Center, Fasa University of Medical Sciences, Fasa, Iran; 6 Department of Pharmaceutics, School of Pharmacy, Pharmaceutical Sciences Research Center, Hemoglobinopathy Institute, Mazandaran University of Medical Sciences, Sari, Iran; 7 Gastroenterohepatology Research Center, Shiraz University of Medical Sciences, Shiraz, Iran; 8 Breast Diseases Research Center, Shiraz University of Medical Sciences, Iran; 9 Non-Communicable Diseases Research Center, Rafsanjan University of Medical Sciences, Rafsanjan, Iran; 10 Genetics of Non-communicable Disease Research Center, Zahedan University of Medical Sciences, Iran; 11 Occupational Medicine Center, Urmia University of Medical Sciences, Iran; 12 Gastrointestinal and Liver Diseases Research Center, Guilan University of Medical Sciences, Rasht, Iran; 13 Digestive Disease Research Center, Ardabil University of Medical Sciences, Ardabil, Iran; 14 Yazd Cardiovascular Research Center, Non-communicable Disease Research Institute, Shahid Sadoughi University of Medical Sciences, Yazd, Iran; 15 Social Determinants of Health Research Center, Research Institute for Health Development, Kurdistan University of Medical Sciences, Sanandaj, Iran; 16 Molecular Medicine Research Center, Hormozgan University of Medical Sciences, Bandar Abbas, Iran; 17 Liver and Gastrointestinal Diseases Research Center, Tabriz University of Medical Sciences, Iran; 18 Nutrition Research Center, Tabriz University of Medical Sciences, Iran; 19 Non-Communicable Diseases Research Center, Department of Environmental Health Engineering, School of Public Health, Sabzevar University of Medical Sciences, Sabzevar, Iran; 20 Department of Internal Medicine, Yasuj University of Medical Sciences, Yasuj, Iran; 21 Behavioral Disease Research Center, Kermanshah University of Medical Sciences, Kermanshah, Iran; 22 Digestive Oncology Research Center, Digestive Diseases Research Institute, Tehran University of Medical Sciences, Tehran, Iran; Muhimbili National Hospital, TANZANIA, UNITED REPUBLIC OF

## Abstract

**Background:**

Dyslipidemia is one of the main risk factors for cardiovascular diseases worldwide. The current study aimed to determine the prevalence and correlates of dyslipidemia in Iran, in addition to factors associated with receiving treatment and attaining treatment goals.

**Methods:**

The current study used the data of a very large cross-sectional survey, Prospective Epidemiological Research Studies in IrAN (PERSIAN), including 163,770 Iranians aged 35–70 years from 18 distinct areas across Iran, between 2014 and 2020. Participants’ characteristics were recorded using validated questionnaires including biological samples, physical measurements, and self-reported information. Dyslipidemia was defined according to the Third Report of the Adult Treatment Panel. Triglycerides (TG), total cholesterol (TC), high-density lipoprotein (HDL) and low-density lipoprotein (LDL) were measured as components of dyslipidemia. We calculated participants’ 10-years cardiovascular risk using the World Health Organization risk charts to assess the eligibility for treatment and achievement of control. Multivariable logistic regression analyses were conducted to investigate the correlates of dyslipidemia, receiving treatment, and control.

**Results:**

The mean age of participants was 49.4 (±9.1) years, among whom 55% were women and 70% were urban residents. The weighted prevalence of dyslipidemia was 73.2% (95% CI: 71.5–75.0%). High TG (40.4%, 36.9%−44.4%) and high TC (36.2%, 32.9%−39.4%) comprised the most prevalent components of dyslipidemia, followed by low HDL-C (32.1%, 25.8%−38.4%), high non-HDL-C (26.6%, 24.0%−29.1%), and high LDL-C (23.4%, 21.1%−25.7%). In multivariable logistic regression analysis, younger age, tobacco consumption, low physical activity, high BMI, diabetes, and hypertension were associated with low HDL-C and high TG. Older age, alcohol consumption, low physical activity, and high BMI were associated with high TC and high LDL-C. Women had higher likelihood of having abnormal levels for all components of dyslipidemia, except for high TG. Older age, high wealth score, high BMI, and low physical activity were associated with higher utilization of statin treatment. Good lipid control in those who were treated with statin was higher in males and diabetics.

**Conclusion:**

The prevalence of dyslipidemia in Iranian population is extremely high, and certain groups are at higher risk of poor lipid control. Findings of this study highlight the necessity of developing a national guideline for management of dyslipidemia.

## Introduction

Dyslipidemia is a disorder in lipid metabolism and a significant contributor to cardiovascular diseases (CVDs). According to the Global Burden of Disease (GBD) latest report, the burden of CVDs continues its steady rise in almost all regions [1]. In 2022, approximately 19.8 million people died from the disease around the world [2]. Dyslipidemia is known as one of the primary risk factors of CVD, which is fortunately preventable and can be simply modified [3]. Considering the heavy economic burden of dyslipidemia [4] and its great contribution to the leading causes of mortality and morbidity worldwide, such as coronary artery disease, type 2 diabetes, and stroke [5], the epidemiology and determinants of lipid abnormalities in different populations are crucial to be investigated. However, large-scale studies in this regard still lack in some low- and middle-income countries (LMICs), including Iran.

Previous studies have shown that over 80% of Iranian adults have at least one lipid abnormality; and the majority do not meet the desired level of LDL-C [6–8]. Also, despite the affordability of lipid-lowering agents, the utilization of such medication is inadequate, which can be a result of poor management and high percentages of undiagnosed patients [9]. Due to the substantial genetic and lifestyle differences in populations, plasma levels of lipids vary among different regions, ages, and genders [10].

During the last decade, several studies have been conducted to investigate the prevalence and correlates of dyslipidemia at the province level in Iran [10–12]. Yet, a clear picture of its nation-wide epidemiology and determinants in different populations across Iran is lacking. Furthermore, most of the Iranian studies conducted by far lack the cardiovascular risk-based approach to dyslipidemia treatment and control. The present study aims to investigate the different patterns of dyslipidemia and their correlates across different ethnicities in Iran, as well as determining level of treatment and control status among those at high risk for developing CVD, using the World Health Organization (WHO) updated guideline for prevention of cardiovascular diseases. We believe this comprehensive study will help policymakers establish more effective control measures to reduce the burden of non-communicable diseases in Iran.

## Methods

### Study setting

The PERSIAN Cohort -standing for Prospective Epidemiological Research Studies in IrAN (PERSIAN) – is a population-based survey launched in 2014 to investigate the epidemiology of non-communicable diseases in the Iranian population [13]. The baseline recruitment phase included 163,770 individuals aged 35–70 years, selected via cluster random sampling from 18 geographically distinct areas across Iran between October 8^th^, 2014 and March 5^th^, 2020. These cohort sites have been chosen to include all the major ethnicities and geographical regions of Iran, comprising areas with low migration rates. Cohort sites were selected from both urban and rural areas based on the following criteria: representation of Iran’s major ethnic groups, inclusion of diverse climates to capture varied environmental and occupational exposures, low migration rates to minimize follow-up loss, and previously reported local disease patterns. Men and women aged 35–70 years residing in the PERSIAN Cohort regions were invited to participate. In smaller cities, all eligible individuals were approached, while in larger cities or multiethnic areas, specific districts were selected to reflect socioeconomic and ethnic diversity. Participants attended the cohort’s recruitment centers, and all eligible individuals were enrolled. Eligibility required Iranian descent and residence in the designated area for at least nine months annually. Individuals with physical or psychological conditions preventing completion of enrollment were excluded. The methodology and design of the cohort have been previously described in detail [14].

### Data collection

To invite the eligible participants, trained fieldworkers went door to door, describing the study objectives and encouraging qualified individuals for participation and referral to the cohort’s recruitment center. Therefore, non-response was not recorded. Upon arrival, study procedures were explained to the participants and written informed consent was obtained from all willing participants before registration. Study procedures included laboratory testing, anthropometric assessment, blood pressure measurement, and questionnaire administration. Bio-specimens were taken from each individual in an overnight 12-hour fasting condition, and included blood, urine, hair, and nail samples. Questionnaires were administered face-to-face by trained interviewers using the standard PERSIAN Cohort instrument, which includes 482 items across general, medical, and nutritional domains. The questionnaire collects detailed sociodemographic, medical, lifestyle, and dietary information, including a validated food-frequency questionnaire. Data were entered online into a centralized database and checked for completeness by field supervisors, and several questionnaire components (e.g., socioeconomic status, physical activity, circadian rhythm, and the FFQ) have been validated within the cohort [14].

Before storing the samples, a small amount of venous blood sample was used for biochemistry tests, including measuring levels of total cholesterol (TC), triglyceride (TG), high density lipoprotein cholesterol (HDL-C), and fasting blood sugar (FBS) using the enzymatic methods [14]. Low-density lipoprotein cholesterol (LDL-C) was calculated using the Friedewald equation [15]. Non-high-density lipoprotein cholesterol (non-HDL-C) was calculated as TC minus HDL-C. Samples were then stored under desired conditions for the duration of follow-up [14]. Anthropometric measurements were performed after sample collection according to the United States National Institutes of Health protocols [16]. Blood pressure (BP) was measured twice from both arms using a mercury sphygmomanometer after 5 minutes of rest in a seated position and with the arm at the heart level [14].

### Definition of variables

In the current study, participants were grouped into four age categories (35–44, 45–54, 55–64, and ≥ 65-year-olds). The residence area was defined as “urban” or “rural”. Education was categorized into three levels of “no schooling” (<1 year of school), “primary school” (1–5 years of school) and “secondary school” (6 years of school or more). Marital status was classified into “married” and “unmarried”. Data on residential property and listed household assets were used to determine the wealth index (WI) using multiple correspondence analysis (MCA). “Economic status” variable was created according to tertiles of WI, and participants were categorized into three economic groups of “low” (poorest tertile), “average”, and “high” (wealthiest tertile). Daily energy expenditure was determined using the Metabolic Equivalent of Tasks (METs, in minute/week), and physical activity was classified as “low,” “moderate,” and “intense” using the MET tertiles. Tobacco consumption was defined as “ever” (consuming any tobacco derivatives, including cigarette, Hookah, Naas or water pipe, at least once a month for the last 6 months) and “never” (otherwise). Opium consumption and alcohol consumption were also divided into “ever” (using at least once a month for the last 6 months) and “never” (if otherwise).

Blood pressure was measured twice in both arms, and systolic blood pressure (SBP) and diastolic blood pressure (DBP) were defined as the mean of second-cycle measurements from the right and left arms, to reduce random measurement variability. Persistent inter-arm differences are known to predict increased cardiovascular risk, and current recommendations advise using the higher arm for clinical decision-making; however, in this epidemiologic analysis the averaged value was used as a continuous exposure measure. Our choice to average is a conscious methodological decision within an epidemiologic context rather than a contradiction of clinical management guidelines. This approach is consistent with the protocol of the PERSIAN study.Hypertension (HTN) was defined as SBP ≥ 140 mmHg, and/or DBP ≥ 90 mmHg [17,18], and/or taking antihypertensive medications. Body Mass Index (BMI) was calculated as weight (kilogram) divided by the square of the height (meter), and categorized according to the protocol of National Institutes of Health in 1998 into groups of “under 25 kg/m^2^”, “25-29.9 kg/m^2^” and “30 kg/m^2^ and higher” [19]. Diabetes was defined according to the American Diabetes Association (ADA) 2020 criteria [20] as FBS ≥ 126 mg/dl, or taking medication for physician-diagnosed diabetes. Established CVD was defined as the positive report of previous coronary heart disease, myocardial infarction, or stroke.

Dyslipidemia was defined according to the Third Report of The National Cholesterol Education Program (NCEP) Expert Panel on Detection, Evaluation, and Treatment of High Blood Cholesterol in Adults (Adult Treatment Panel III) [21], as having any of the following criteria: (1) TC ≥ 200 mg/dL; (2) TG ≥ 150 mg/dL; (3) LDL-C ≥ 130 mg/dL; (4) HDL-C < 40 mg/dL in men and <50 mg/dL in women; (5) taking any kind of lipid regulating drugs, including statins and non-statins (fibrates, niacin and bile acid resins). As an additive measure, high non-HDL-C was defined as non-HDL-C ≥ 160 mg/dL.

Eligibility of treatment with statin medications and control of high cholesterol were defined according to the WHO guidelines [22,23]. We calculated individuals’ 10-year CVD risk using the updated (2019) laboratory-based WHO cardiovascular disease risk charts for the North African and Middle East region (Iran) [23]. These charts use the data on age, sex, smoking status, SBP, presence of diabetes, and TC, to calculate sex-specific 10-year risk of a cardiovascular outcome, defined as myocardial infarction, coronary heart disease, or stroke.

The following four groups were considered eligible for receiving statin treatment according to WHO Package of Essential Non-communicable (PEN) disease intervention for primary health care (2020) [24]: (1) previously established cardiovascular disease; (2) ≥ 20% WHO 10-year CVD risk; (3) TC ≥ 320 mg/dl, or LDL-C ≥ 240 mg/dl, or TC/LDL-C ratio>8; (4) Diabetics over 40 years of age; and (5) patients with known renal failure. We considered patients with a history of cardiovascular disease as “established CVD”, while the other eligible groups of individuals were considered as “high risk”. Control of cholesterol was defined according to 2007 WHO guideline for prevention of cardiovascular disease in high and very high-risk groups. Participants with established CVD were considered to have good lipid control if they had TC < 152 mg/dL and LDL-C < 77 mg/dL while receiving statin therapy. For individuals classified as high risk, good lipid control was defined as LDL-C < 115 mg/dL (3.0 mmol/L) and total cholesterol < 190 mg/dL (5.0 mmol/L) while receiving statin. For very high–risk individuals, good lipid control was defined as LDL-C < 77 mg/dL (2.0 mmol/L) and total cholesterol < 152 mg/dL (4.0 mmol/L), in accordance with guideline recommendations. [22] Participants were defined as receiving statin if they reported taking any dosages of statin medications (including atorvastatin, lovastatin, simvastatin, somatostatin, rosuvastatin) for the past three months.

### Data quality and handling

The PERSIAN Cohort implemented standardized data quality procedures, including automated range checks, internal consistency validation (e.g., BMI plausibility based on height/weight), and centralized data monitoring by field supervisors. Outlier detection and data cleaning were performed at the baseline phase of the PERSIAN cohort ahead of the launch of the current secondary study on cohort data. Biologically implausible lipid values (e.g., TG > 2000 mg/dL, TC > 500 mg/dL) and blood pressure outliers (>300/200 mmHg) were flagged during data entry and reviewed by site coordinators prior to analysis. Affected records (<0.1% of total) were corrected or excluded after source verification.

### Statistical analysis

Given the cluster sampling design of the PERSIAN cohort, we analyzed all data using survey methods. We used a complex survey design to obtain summary measures. We used sampling weights defined as the inverse probability of being selected in the survey based on data of the national census in 2016, to obtain weighted estimates that reflect the underlying target population. We did not perform additional post-stratification adjustments beyond those incorporated in the PERSIAN Cohort design.

We first described baseline characteristics of participants overall and by dyslipidemia status.

We assessed the distribution of lipid parameters (TC, LDL-C, HDL-C, non-HDL-C and TG) using distributional diagnostic plots, and reported the values as median (quartile 25, quartile 75), since they all showed non-normal distribution. Weighted prevalence of different dyslipidemia components is presented as percentages, with their 95% confidence intervals (95% CI) across categorical variables.

For regression analyses, we used weighted multivariable logistic regression models to estimate odds ratios (ORs) and 95% CIs for the associations between covariates and each dyslipidemia component. Separate models were fitted for: (1) correlates of dyslipidemia components, (2) correlates of statin use among “high-risk” and “established CVD” groups, and (3) correlates of good lipid control among all eligible participants and among those receiving statins. All models included age category, sex, residence area, education, wealth index category, marital status, physical activity, BMI category, alcohol consumption, tobacco consumption, opium use, diabetes, hypertension, and ethnicity. Tobacco use, diabetes, and hypertension were not entered in high-risk models because they contribute to the estimated WHO CVD risk score.

To account for clustering and stratification, we specified the complex survey design in Stata using the *svyset* command with primary sampling units, strata, and sampling weights, and used survey-adjusted logistic regression for all inferential analyses. Potential multi-collinearity was assessed using variance inflation factors, which didn’t exceed a threshold of 5 indicating absence of collinearity. Overall model calibration was evaluated using the Hosmer–Lemeshow goodness-of-fit test in unweighted models, which indicated adequate fit. We additionally explored sex-by-age interaction terms for overall dyslipidemia and key lipid components.

Missing lipid profiles led to the exclusion of 2,008 participants (1.2%). We compared baseline characteristics of excluded and included participants and found similar distributions of key variables, suggesting minimal risk of selection bias.

All analyses were conducted using Stata Statistical Software, Release 12 (StataCorp, College Station, TX), which provides the survey commands used in this study. Statistical significance was defined as a two-sided P-value <0.05, without formal adjustment for multiple testing. This is noted as a limitation, and results are interpreted considering consistency and magnitude of associations rather than P-values alone.

### Ethics approval

Iranian Ministry of Health and Medical Education supervises the project, and investigators at medical universities are responsible for the conduct of this project in each cohort site. The design of the PERSIAN Cohort study was approved by the ethics committees of the Tehran University of Medical Sciences, the Digestive Diseases Research Institute (IR.TUMS.DDRI.REC.1392.001).

## Results

### Baseline characteristics

The current study included 161,762 participants in the PERSIAN Cohort Study, of whom 44.8% were men. The mean age of all participants was 49.39 (±9.14) years, and 64.6% aged 45 years or older. More than 70% of all participants resided in urban areas, and Fars comprised the most common ethnicity (30.1%). Overall, 14,838 (9.2%) had a CVD history (Table 1).

**Table 1:** Baseline characteristics of the PERSIAN Cohort Study participants.

		Without dyslipidemia	With dyslipidemia	Overall
		Number (%)	Number (%)	Number (%)
**Total**		**39,816 (100)**	**121,946 (100)**	**161,762 (100)**
**Gender**	**Men**	21,238 (53.3)	51,179 (42.0)	72,417 (44.8)
	**Women**	18,578 (46.7)	70,767 (58.0)	89,345 (55.2)
**Age**	**35-44 years**	16,658 (41.8)	40,605 (33.3)	57,263 (35.4)
	**45-54 years**	12,760 (32.1)	42,045 (34.5)	54,805 (33.9)
	**55-64 years**	8,159 (20.5)	31,132 (25.5)	39,291 (24.3)
	**≥65 years**	2,239 (5.6)	8,164 (6.7)	10,403 (6.4)
**Residence area**	**Urban**	26,765 (67.2)	87,735 (72.0)	114,500 (70.8)
	**Rural**	13,051 (32.8)	34,211 (28.0)	47,262 (29.2)
**Education**	**0 years**	7,128 (17.9)	26,133 (21.4)	33,261 (20.6)
	**1-5 years**	12,349 (31.1)	39,090 (32.1)	51,439 (31.8)
	**6 and more years**	20,293 (51.0)	56,610 (46.5)	76,903 (47.6)
**Wealth Score index category**	**Low**	14,007 (35.3)	41,212 (33.9)	55,219 (34.2)
	**Average**	12,829 (32.4)	39,639 (32.6)	52,468 (32.6)
	**High**	12,819 (32.3)	40,701 (33.5)	53,520 (33.2)
**Marital status**	**Unmarried**	3,009 (7.6)	11,420 (9.4)	14,429 (8.9)
	**Married**	36,807 (92.4)	110,526 (90.6)	147,333 (91.1)
**Tobacco consumption**	**Never**	27,867 (70.3)	90,340 (74.3)	118,207 (73.3)
	**Ever**	11,762 (29.7)	31,180 (25.7)	42,942 (26.7)
**Opium consumption**	**Never**	34,813 (87.4)	111,071 (91.1)	145,884 (90.2)
	**Ever**	5,003 (12.6)	10,875 (8.9)	15,878 (9.8)
**Alcohol consumption**	**Never**	36,837 (92.9)	114,033 (93.8)	150,870 (93.6)
	**Ever**	2,794 (7.1)	7,496 (6.2)	10,290 (6.4)
**Physical activity**	**Low**	11,238 (28.4)	42,477 (35.0)	53,715 (33.4)
	**Average**	12,320 (31.1)	41,390 (34.0)	53,710 (33.3)
	**High**	16,070 (40.5)	37,640 (31.0)	53,710 (33.3)
**BMI**	**<25**	17,325 (43.7)	27,332 (22.5)	44,657 (27.7)
	**25-29.9**	14,111 (35.6)	51,602 (42.5)	65,713 (40.8)
	**≥30**	8,201 (20.7)	42,585 (35.0)	50,786 (31.5)
**Diabetes**	**No**	37,240 (93.5)	101,993 (83.6)	139,233 (86.1)
	**Yes**	2,576 (6.5)	19,953 (16.4)	22,529 (13.9)
**Hypertension**	**No**	28,673 (72.0)	72,230 (59.2)	100,903 (62.4)
	**Yes**	11,143 (28.0)	49,716 (40.8)	60,859 (37.6)
**Cardiovascular disease**	**No**	37,494 (94.6)	108,880 (89.6)	146,374 (90.8)
	**Yes**	2,151 (5.4)	12,687 (10.4)	14,838 (9.2)

Abbreviations: BMI, Body Mass Index.

### Prevalence of dyslipidemia and lipid components

High TG (40.4%; 95% CI: 36.9–44.4) and high TC (36.2%; 95% CI: 32.9–39.4) comprised the most prevalent components of dyslipidemia in our study, followed by low HDL-C (32.1%; 95% CI: 25.8–38.4) and high LDL-C (23.4%; 95% CI: 21.1–25.7). High non-HDL-C was observed in 26.6% (95% CI: 24.0–29.1) of individuals (Table 2). The median (quartile 25, quartile 75) of TC, LDL-C, HDL-C, TG and non-HDL-C were 187.5 (163, 215) mg/dl, 108.2 (87.6, 130.6) mg/dl, 48.7 (41, 57) mg/dl, 130 (94.5, 185) mg/dl and 114.4 (138, 163) mg/dl, respectively (S1 Table).

**Table 2:** Weighted prevalence of different components of dyslipidemia and associated factors in multivariable regression analysis models*.

		TC ≥ 200 mg/dL		LDL-C ≥ 130 mg/dL		HDL-C < 40 mg/dL in men and <50 mg/dL in women		TG ≥ 150 mg/dL		Non-HDL-C ≥ 160 mg/dL	
		% (95% CI)	OR (95% CI)	% (95% CI)	OR (95% CI)	% (95% CI)	OR (95% CI)	% (95% CI)	OR (95% CI)	% (95% CI)	OR (95% CI)
**Total**		**36.2 (32.9-39.4)**	**–**	**23.4 (21.1-25.7)**	**–**	**32.1 (25.8-38.4)**	**–**	**40.4 (36.9-44.4)**	**–**	**26.6 (24.0-29.1)**	**–**
**Gender**	**Men**	32.9 (29.7-36.1)	*Reference*	21.0 (18.8-23.1)	*Reference*	24.7 (19.0-30.3)	*Reference*	44.5 (39.9-49.0)	*Reference*	26.1 (23.8-28.6)	*Reference*
	**Women**	39.5 (36.0-43.0)	**1.20 (1.12-1.29)**	25.9 (23.1-28.7)	**1.22 (1.11-1.34)**	39.8 (33.0-46.7)	**2.05 (1.85-2.26)**	36.2 (32.6-39.8)	**0.56 (0.52-0.61)**	27.0 (24.1-29.9)	0.93 (0.85-1.00)
**Age**	**35-44 years**	30.3 (27.0-33.5)	*Reference*	19.2 (17.1-21.2)	*Reference*	34.7 (28.2-41.1)	*Reference*	37.1 (33.0-41.2)	*Reference*	22.1 (19.8-24.4)	*Reference*
	**45-54 years**	39.7 (35.8-43.6)	**1.52 (1.43-1.61)**	25.4 (22.7-28.1)	**1.47 (1.35-1.60)**	31.2 (24.5-37.9)	**0.77 (0.71-0.82)**	43.7 (40.0-47.4)	1.12 (1.00-1.18)	29.3 (26.3-32.3)	**1.41 (1.31-1.51)**
	**55-64 years**	42.5 (39.6-45.5)	**1.75 (1.60-1.92)**	28.4 (25.5-31.2)	**1.78 (1.60-1.99)**	29.1 (23.0-35.2)	**0.62 (0.56-0.69)**	42.8 (38.3-47.3)	0.98 (0.94-1.02)	31.5 (28.7-34.3)	**1.56 (1.42-1.72)**
	**≥65 years**	39.8 (36.7-42.9)	**1.59 (1.38-1.83)**	27.4 (23.9-30.8)	**1.70 (1.45-1.99)**	28.3 (24.2-32.4)	**0.54 (0.48-0.62)**	40.2 (35.9-44.5)	**0.84 (0.75-0.94)**	29.0 (25.6-32.4)	**1.37 (1.15-1.62)**
**Residence area**	**Urban**	37.0 (33.2-40.8)	*Reference*	23.7 (20.9-26.5)	*Reference*	31.6 (23.9-39.3)	*Reference*	42.0 (37.6-46.4)	*Reference*	27.3 (24.3-30.2)	*Reference*
	**Rural**	33.3 (29.1-37.4)	0.88 (0.72-1.08)	22.3 (18.6-26.1)	0.90 (0.73-1.10)	33.8 (26.1-41.6)	1.03 (0.62-1.73)	35.0 (29.6-40.4)	0.87 (0.64-1.16)	24.1 (20.2-27.9)	0.86 (0.73-1.01)
**Education**	**0 years**	42.1 (38.5-45.7)	*Reference*	59.0 (26.8-31.3)	*Reference*	36.0 (30.3-41.4)	*Reference*	39.0 (33.9-44.1)	*Reference*	30.8 (27.8-33.9)	*Reference*
	**1-5 years**	36.4 (32.4-40.5)	**0.84 (0.79-0.90)**	23.9 (21.3-26.5)	**0.88 (0.82-0.94)**	34.7 (28.3-41.2)	1.02 (0.95-1.09)	38.8 (33.9-43.6)	**0.88 (0.79-0.97)**	26.5 (23.3-29.6)	**0.84 (0.79-0.89)**
	**6 and more years**	34.5 (31.4-37.6)	**0.85 (0.79-0.92)**	21.7 (19.4-24.0)]	**0.88 (0.80-0.97)**	29.8 (23.4-36.2)	0.95 (0.86-1.05)	41.6 (38.1-45.0)	**0.87 (0.79-0.95)**	25.5 (23.2-27.8)	**0.84 (0.78-0.91)**
**Wealth Score index category**	**Low**	35.1 (31.2-38.9)	*Reference*	24.4 (22.1-26.6)	*Reference*	36.1 (30.8-41.4)	*Reference*	35.1 (30.6-39.7)	*Reference*	25.9 (22.8-28.9)	*Reference*
	**Average**	35.6 (32.6-38.6)	1.04 (0.99-1.10)	22.6 (20.4-24.8)	0.96 (0.91-1.01)	32.3 (25.9-38.7)	0.92 (0.77-1.11)	40.4 (36.6-44.1)	**1.09 (1.01-1.18)**	26.1 (23.8-28.4)	1.00 (0.95-1.05)
	**High**	37.5 (34.2-40.8)	**1.10 (1.03-1.19)**	23.4 (20.4-36.4)	1.00 (0.90-1.10)	29.1 (22.3-35.9)	0.84 (0.64-1.10)	44.1 (40.9-47.4)	**1.16 (1.04-1.30)**	27.5 (24.5-30.5)	1.02 (0.95-1.10)
**Marital status**	**Unmarried**	41.2 (36.5-45.9)	*Reference*	28.1 (25.1-31.1)	*Reference*	37.2 (30.5-43.9)	*Reference*	36.9 (32.4-41.4)	*Reference*	29.5 (25.8-33.2)	*Reference*
	**Married**	35.8 (32.6-38.9)	**0.88 (0.81-0.95)**	23.0 (20.7-25.3)	**0.89 (0.83-0.96)**	31.7 (25.5-37.9)	1.06 (0.96-1.17)	40.7 (36.7-44.7)	**0.94 (0.90-0.98)**	26.3 (23.9-28.8)	**0.87 (0.81-0.94)**
**Tobacco consumption**	**Never**	37.4 (34.1-40.7)	*Reference*	24.3 (21.8-26.8)	*Reference*	33.7 (27.5-39.9)	*Reference*	39.0 (35.3-42.7)	*Reference*	26.6 (23.9-29.3)	*Reference*
	**Ever**	33.1 (29.9-36.3)	0.99 (0.96-1.04)	21.1 (19.2-23.1)	0.98 (0.94-1.02)	28.3 (21.6-35.0)	**1.25 (1.12-1.39)**	43.8 (38.5-49.0)	**1.09 (1.03-1.16)**	26.5 (24.0-28.9)	**1.06 (1.01-1.12)**
**Opium consumption**	**Never**	36.9 (33.7-40.2)	*Reference*	23.9 (21.5-26.4)	*Reference*	32.8 (26.4-39.1)	*Reference*	40.3 (36.3-44.2)	*Reference*	26.9 (24.2-29.6)	*Reference*
	**Ever**	30.4 (27.3-33.5)	**0.90 (0.84-0.97)**	19.2 (17.7-20.7)	**0.88 (0.80-0.98)**	27.2 (19.2-35.2)	1.04 (0.87-1.24)	41.5 (35.6-47.5)	1.02 (0.92-1.12)	24.3 (22.3-26.2)	0.95 (0.86-1.04)
**Alcohol consumption**	**Never**	36.2 (32.9-39.6)	*Reference*	23.5 (21.1-25.9)	*Reference*	32.8 (26.4-39.1)	*Reference*	39.8 (35.9-43.8)	*Reference*	26.4 (23.8-29.0)	*Reference*
	**Ever**	35.6 (32.9-38.3)	**1.19 (1.11-1.29)**	22.6 (20.1-25.0)	**1.19 (1.07-1.32)**	24.7 (18.8-30.6)	0.90 (0.80-1.01)	47.4 (42.6-52.2)	**1.09 (1.01-1.17)**	28.8 (26.4-31.2)	**1.18 (1.09-1.27)**
**Physical activity**	**Low**	37.3 (33.7-40.9)	*Reference*	24.2 (21.9-26.6)	*Reference*	34.3 (26.8-41.9)	*Reference*	45.8 (40.9-50.6)	*Reference*	28.6 (26.1-31.1)	*Reference*
	**Average**	38.0 (34.1-41.8)	0.98 (0.93-1.04)	24.4 (21.5-27.5)	0.97 (0.91-1.04)	33.8 (26.7-40.9)	**0.90 (0.84-0.96)**	40.6 (36.8-44.4)	**0.90 (0.86-0.94)**	27.5 (24.5-30.4)	**0.96 (0.91-1.02)**
	**High**	33.7 (30.8-36.7)	**0.91 (0.87-0.96)**	21.9 (19.8-24.0)	**0.92 (0.88-0.96)**	28.9 (23.7-34.1)	**0.82 (0.73-0.93)**	35.8 (31.5-40.1)	**0.72 (0.66-0.79)**	24.2 (21.6-26.7)	**0.85 (0.81-0.88)**
**BMI**	**<25**	27.5 (24.9-30.1)	*Reference*	19.0 (17.1-20.9)	*Reference*	25.2 (19.3-31.1)	*Reference*	24.9 (21.8-28.0)	*Reference*	18.3 (16.4-20.2)	*Reference*
	**25-29.9**	37.9 (35.4-40.4)	**1.50 (1.44-1.56)**	24.2 (22.0-26.5)	**1.29 (1.23-1.36)**	32.8 (26.1-39.5)	**1.42 (1.35-1.48)**	44.2 (40.9-47.5)	**2.38 (2.21-2.55)**	28.6 (26.7-30.4)	**1.72 (1.63-1.82)**
	**≥30**	41.8 (37.9-45.7)	**1.65 (1.55-1.75)**	26.3 (23.4-29.2)	**1.34 (1.27-1.42)**	37.5 (30.1-44.9)	**1.49 (1.38-1.61)**	49.5 (45.6-53.5)	**3.14 (2.77-3.55)**	31.4 (28.1-34.7)	**1.92 (1.80-2.05)**
**Diabetes**	**No**	36.0 (32.6-39.3)	*Reference*	23.8 (21.4-26.2)	*Reference*	31.5 (25.4-37.5)	*Reference*	37.8 (33.8-41.8)	*Reference*	26.1 (23.5-28.8)	*Reference*
	**Yes**	37.5 (34.6-40.4)	**0.86 (0.80-0.92)**	20.8 (18.7-23.0)	**0.69 (0.64-0.74)**	36.4 (28.3-44.4)	**1.23 (1.10-1.37)**	57.2 (53.3-61.1)	**1.93 (1.80-2.06)**	29.3 (27.0-31.5)	0.96 (0.90-1.03)
**Hypertension**	**No**	34.7 (30.7-38.7)	*Reference*	22.4 (19.9-24.9)	*Reference*	30.6 (24.6-36.5)	*Reference*	37.1 (32.4-41.9)	*Reference*	24.9 (21.7-28.2)	*Reference*
	**Yes**	39.0 (36.0-41.9)	1.07 (1.00-1.15)	25.3 (22.6-27.8)	1.07 (0.99-1.16)	35.1 (28.2-41.9)	**1.21 (1.12-1.32)**	46.7 (42.2-51.2)	**1.23 (1.14-1.33)**	29.7 (27.4-32.0)	**1.12 (1.04-1.21)**
**Cardiovascular disease**	**No**	36.7 (33.4-40.0)	*Reference*	23.8 (21.4-26.1)	*Reference*	31.8 (25.6-38.1)	*Reference*	40.0 (36.0-44.0)	*Reference*	26.9 (24.4-29.4)	*Reference*
	**Yes**	30.4 (26.9-33.9)	**0.59 (0.53-0.65)**	19.3 (16.5-22.1)	**0.63 (0.58-0.69)**	36.2 (29.3-43.0)	**1.23 (1.11-1.37)**	44.3 (39.6-48.9)	**0.91 (0.84-0.99)**	22.8 (19.9-25.7)	**0.62 (0.57-0.68)**

Abbreviations:: TC, Total cholesterol; LDL-C, Low-density lipoprotein cholesterol; HDL-C, High-density lipoprotein cholesterol; TG, Triglyceride; CI, Confidence Interval; OR, Odds Ratio; BMI, Body Mass Index

*ORs were adjusted for all variables in the column as well as ethnicity.

**Fig 1:**
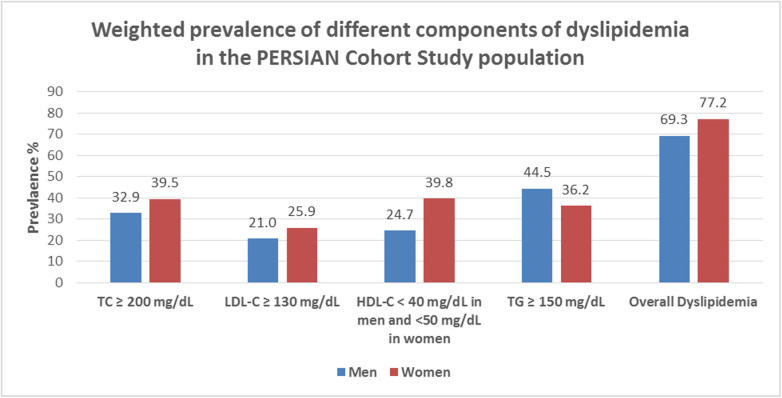
Weighted prevalence of different components of dyslipidemia in the PERSIAN Cohort Study population. Weighted prevalence (%) of each dyslipidemia component in the study population. Estimates are based on standardized biochemical measurements; abbreviations include TC, TG, LDL-C, HDL-C, and non-HDL-C.

**Fig 2:**
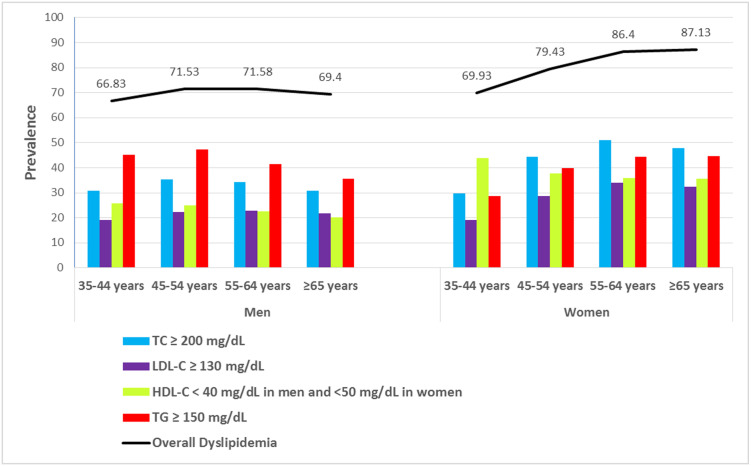
Weighted prevalence of different components of dyslipidemia in different age and sex groups. Weighted prevalence (%) of dyslipidemia components stratified by age group and sex. Component definitions and weighting methods follow the study protocol.

The weighted prevalence of overall dyslipidemia was 73.2% (95% CI: 71.5–75.0), which was lower in men (69.4%; 95% CI: 67.7–71.2) than women (77.2%; 95% CI: 75.5–79.0) (P-value<0.001). High TC and LDL-C, and low HDL-C were higher in women than men, while high TG was more prevalent in men (P-value<0.001) (Fig 1). While dyslipidemia prevalence was steady across age groups in men, the prevalence of all lipid abnormalities (except for low HDL-C) increased with age in women (Fig 2).

### Correlates of dyslipidemia

In multivariable regression analysis, younger age, low education, tobacco consumption, low physical activity, high BMI, diabetes and hypertension were associated with low HDL-C and high TG. Ethnicities of Balouch (OR: 2.64; 95% CI: 1.74–3.99) and Kurd (OR: 2.19; 95% CI: 1.31–3.68) had higher odds for low HDL-C, but lower odds for high TC (OR: 0.68; 95% CI: 0.60–0.78 and OR: 0.77; 95% CI: 0.61–0.96, respectively) compared to Fars ethnicity as the reference. Other ethnicities showed no association with dyslipidemia. Furthermore, older age, low education, being unmarried, alcohol consumption, low physical activity, and high BMI were associated with high TC, high LDL-C and high non-HDL-C, while diabetes and cardiovascular disease had lower odds for high TC and high LDL-C. Female sex had higher likelihood for all components of lipid abnormalities, except for high TG (OR: 0.56, 95% CI: 0.52–0.61). Opium consumption was inversely associated with both high TC (OR: 0.90; 95% CI: 0.84–0.97) and high LDL-C (OR: 0.88; 95% CI: 0.80–0.98), while tobacco consumption was associated with low HDL-C (OR: 1.25, 95% CI: 1.12–1.39) and high TG (OR: 1.09, 95% CI: 1.03–1.16). Age had a direct association with overall dyslipidemia in women (OR for the oldest versus the youngest: 2.05 [95% CI: 1.80–2.34]), while having an inverse association in men (OR: 0.68 [95% CI: 0.61–0.76]) (Table 3). The positive association of female sex with increasing age remained significant even after adjusting the model for menopause (p-for-trend<0.001) S2 Table).

**Table 3:** Weighted multivariable logistic regression analysis* of factors associated with dyslipidemia stratified by sex.

		All	Sex stratification	
		OR (95% CI)	Men	Women
			OR (95% CI)	OR (95% CI)
**Gender**	**Men**	*Reference*	**–**	**–**
	**women**	**0.58 (0.54-0.63)**	–	–
**Age**	**35-44 years**	*Reference*	*Reference*	*Reference*
	**45-54 years**	**1.15 (1.12-1.19)**	0.98 (0.92-1.04)	**1.43 (1.32-1.54)**
	**55-64 years**	**1.24 (1.17-1.31)**	**0.86 (0.80-0.92)**	**1.97 (1.76-2.20)**
	**≥65 years**	**1.10 (1.02-1.19)**	**0.68 (0.61-0.76)**	**2.05 (1.80-2.34)**
**Residence area**	**Urban**	*Reference*	*Reference*	*Reference*
	**Rural**	**0.87 (0.78-0.98)**	0.89 (0.74-1.07)	**0.86 (0.77-0.96)**
**Education**	**0 years**	*Reference*	*Reference*	*Reference*
	**1-5 years**	**0.91 (0.84-0.99)**	1.03 (0.96-1.11)	0.99 (0.92-1.08)
	**≥ 6 years**	**0.87 (0.81-0.94)**	1.02 (0.92-1.13)	0.92 (0.85-1.01)
**Wealth Score index category**	**Low**	*Reference*	*Reference*	*Reference*
	**Average**	1.04 (0.98-1.11)	1.07 (0.99-1.16)	1.03 (0.97-1.09)
	**High**	1.02 (0.92-1.12)	1.07 (0.93-1.22)	0.98 (0.91-1.06)
**Marital status**	**Unmarried**	*Reference*	*Reference*	*Reference*
	**Married**	**0.90 (0.85-0.95)**	1.10 (0.92-1.31)	0.96 (0.90-1.03)
**Tobacco consumption**	**Never**	*Reference*	*Reference*	*Reference*
	**Ever**	**1.12 (1.04-1.20)**	**1.15 (1.06-1.24)**	1.07 (0.99-1.16)
**Opium consumption**	**Never**	*Reference*	*Reference*	*Reference*
	**Ever**	0.99 (0.88-1.11)	0.99 (0.88-1.12)	1.12 (0.98-1.29)
**Alcohol consumption**	**Never**	*Reference*	*Reference*	*Reference*
	**Ever**	**1.08 (1.01-1.17)**	1.03 (0.96-1.10)	1.05 (0.63-1.74)
**Physical activity**	**Low**	*Reference*	*Reference*	*Reference*
	**Average**	**0.89 (0.85-0.93)**	**0.90 (0.85-0.94)**	**0.91 (0.86-0.96)**
	**High**	**0.73 (0.69-0.77)**	**0.72 (0.67-0.77)**	**0.76 (0.70-0.82)**
**BMI**	**<25**	*Reference*	*Reference*	*Reference*
	**25-29.9**	**1.86 (1.78-1.93)**	**2.01 (1.90-2.13)**	**1.58 (1.48-1.67)**
	**≥30**	**2.26 (2.07-2.46)**	**2.40 (2.14-2.69)**	**1.95 (1.83-2.07)**
**Diabetes**	**No**	*Reference*	*Reference*	*Reference*
	**Yes**	**2.50 (2.34-2.66)**	**2.10 (1.90-2.33)**	**2.78 (2.60-2.96)**
**Hypertension**	**No**	*Reference*	*Reference*	*Reference*
	**Yes**	**1.34 (1.26-1.43)**	**1.32 (1.26-1.39)**	**1.30 (1.19-1.42)**
**Cardiovascular disease**	**No**	*Reference*	*Reference*	*Reference*
	**Yes**	**1.93 (1.79-2.08)**	**2.41 (2.16-2.70)**	**1.58 (1.46-1.71)**

Abbreviations: OR, Odds Ratio; CI, Confidence Interval; BMI, Body Mass Index

* ORs were adjusted for all variables in the column as well as ethnicity

Overall, higher BMI, lower education, and lower physical activity showed a consistent pattern of association with most dyslipidemia components, and detailed ORs are presented in Table 2.

### Statin eligibility, treatment, and lipid control

**Fig 3:**
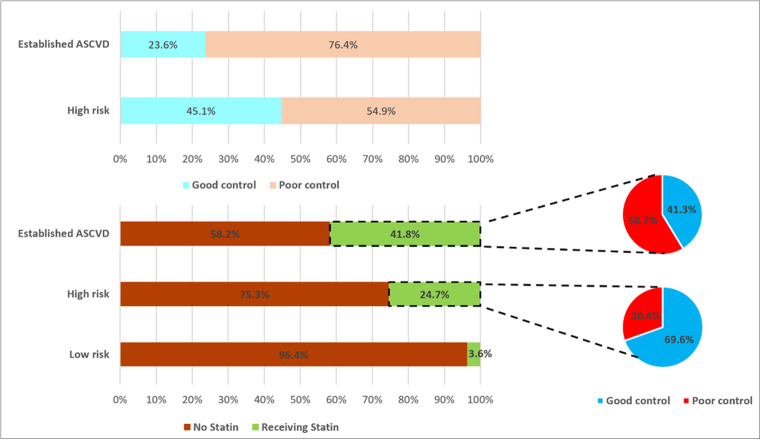
The percentage of lipid control among different ASCVD risk groups. **ASCVD: Atherosclerotic Cardiovascular Disease.** Percentage of participants achieving lipid-control targets within each ASCVD risk group. Definitions of lipid-control thresholds and ASCVD categories follow the study protocol.

A total of 34,884 participants (weighted prevalence: 19.8% (95% CI: 17.9%−21.6%) of the population) belonged to either “high risk” (11.6%, 9.9%−13.2%) or “established CVD” (8.2%, 7.5%−8.9%) group, and had indication for starting statin, regardless of their LDL-C level. Among “high risk” and “established CVD” individuals, only 45.1% (42.0%−48.2%) and 23.6% (20.5%−26.7%) had achieved good control of LDL-C and TC. In the “high risk” group, only 24.7% received statin, of whom, 69.6% (67.7%−71.4%) had good control. Among individuals with “established CVD”, only 41.8% were receiving statin, 41.3% (38.4%−44.3%) of whom achieved lipid goals (Fig 3).

### Determinants of statin use and lipid control

In multivariable logistic regression analysis, female sex was positively associated with statin use (OR: 2.41 [95% CI: 2.14–2.72] in high-risk group, while the association was negative in the group with established CVD (0.82 [0.69–0.98]). Older age was also associated with statin treatment in the high risk (OR: 1.92 [1.6–2.29]) and “established CVD” groups (OR: 3.57 [2.74–4.64]), and p-for-trend was < 0.001 in both groups. Urban residence, high wealth score, high BMI, low physical activity and opium consumption were associated with statin treatment in the “high risk” group. High education, high wealth score, low physical activity, and high BMI were also associated with statin treatment in “established CVD” group. In those with “established CVD”, opium consumption, diabetes and hypertension were associated with higher odds of being under treatment.

Among all high-risk eligible participants (treated and untreated), good lipid control was associated with higher education, and not drinking alcohol. Good control was associated with male sex, older age, higher education, being married, low physical activity, and having diabetes and hypertension in eligible individuals with CVD (Table 4).

**Table 4:** Weighted multivariable logistic regression analysis* of factors associated with statin treatment and LDL-C control, among participants with high and very high WHO 10-year risk of ASCVD.

		OR (95% CI)					
		Receiving statin in all eligible individuals		Good control** in all eligible individuals		Good control in treated eligible individuals	
		High risk (n = 19,268)	Established ASCVD (n = 14,833)	High risk (n = 19,268)	Established ASCVD (n = 14,833)	High risk (n = 4,689)	Established ASCVD (n = 5,821)
**Gender**	**Men**	*Reference*					
	**Women**	**2.41 (2.14-2.72)**	**0.82 (0.69-0.98)**	0.93 (0.86-1.01)	**0.51 (0.45-0.59)**	**0.67 (0.60-0.76)**	**0.43 (0.34-0.55)**
**Age**	**35-44 years**	*Reference*					
	**45-54 years**	**1.76 (1.51-2.06)**	**2.72 (2.02-3.65)**	1.10 (0.95-1.28)	1.15 (0.92-1.45)	1.09 (0.82-0.1.44)	1.13 (0.81-1.57)
	**55-64 years**	**2.34 (2.08-2.63)**	**3.42 (2.79-4.18)**	1.11 (0.94-1.31)	1.25 (0.98-1.61)	1.08 (0.76-1.54)	1.09 (0.72-1.64)
	**≥65 years**	**1.92 (1.60-2.29)**	**3.57 (2.74-4.64)**	1.06 (0.83-1.35)	**1.51 (1.09-2.08)**	**1.35 (1.01-1.80)**	1.28 (0.82-2.00)
**Residence area**	**Urban**	*Reference*					
	**Rural**	**0.66 (0.58-0.76)**	0.86 (0.69-1.07)	0.93 (0.80-1.10)	0.97 (0.79-1.18)	0.87 (0.72-1.04)	0.92 (0.74-1.15)
**Education**	**0 years**	*Reference*					
	**1-5 years**	0.98 (0.87-1.10)	1.08 (0.96-1.23)	**1.20 (1.08-1.34)**	**1.24 (1.05-1.47)**	1.07 (0.93-1.22)	1.17 (0.96-1.44)
	**≥ 6 years**	0.98 (0.85-1.13)	**1.21 (1.09-1.34)**	**1.18 (1.02-1.36)**	**1.15 (1.01-1.33)**	1.14 (0.82-1.58)	0.98 (0.76-1.26)
**Wealth Score index**	**Low**	*Reference*					
	**Average**	**1.15 (1.03-1.27)**	**1.21 (1.10-1.34)**	1.05 (0.95-1.15)	1.04 (0.89-1.23)	0.90 (0.75-1.07)	0.88 (0.68-1.14)
	**High**	**1.18 (1.06-1.33)**	**1.32 (1.14-1.53)**	1.03 (0.93-1.15)	1.17 (0.95-1.43)	1.00 (0.83-1.21)	0.99 (0.74-1.32)
**Marital status**	**Unmarried**	*Reference*					
	**Married**	0.97 (0.87-1.08)	0.93 (0.74-1.20)	1.09 (0.93-1.28)	**1.28 (1.02-1.61)**	1.11 (0.86-1.45)	1.39 (0.97-1.98)
**Tobacco consumption**	**Never**	*Reference*					
	**Ever**	NA***	0.99 (0.83-1.18)	NA	1.09 (0.97-1.23)	NA	1.09 (0.97-1.23)
**Opium consumption**	**Never**	*Reference*					
	**Ever**	**0.85 (0.73-0.99)**	**0.79 (0.63-0.99)**	1.03 (0.89-1.20)	0.91 (0.79-1.04)	1.33 (0.99-1.80)	0.88 (0.73-1.05)
**Alcohol consumption**	**Never**	*Reference*					
	**Ever**	1.03 (0.81-1.31)	**1.25 (1.01-1.55)**	**0.83 (0.70-0.99)**	0.88 (0.70-1.11)	0.72 (0.36-1.45)	0.83 (0.65-1.06)
**Physical activity**	**Low**	*Reference*					
	**Average**	**0.86 (0.80-0.93)**	**0.86 (0.78-0.95)**	0.93 (0.87-1.00)	**0.87 (0.79-0.96)**	1.01 (0.79-1.29)	0.91 (0.78-1.06)
	**High**	**0.80 (0.68-0.94)**	**0.80 (0.71-0.91)**	**0.89 (0.81-0.99)**	0.92 (0.73-1.16)	0.94 (0.77-1.15)	0.99 (0.77-1.29)
**BMI**	**<25**	*Reference*					
	**25-29.9**	**1.27 (1.15-1.42)**	**1.23 (1.10-1.37)**	0.96 (0.87-1.06)	0.93 (0.73-1.18)	1.07 (0.81-1.42)	1.11 (0.88-1.41)
	**≥30**	**1.41 (1.32-1.51)**	**1.31 (1.13-1.52)**	0.90 (0.82-1.00)	0.99 (0.80-1.24)	1.05 (0.78-1.43)	1.07 (0.81-1.42)
**Diabetes**	**No**	*Reference*					
	**Yes**	NA	**2.12 (1.91-2.36)**	NA	**1.61 (1.44-1.79)**	NA	**1.23 (1.03-1.46)**
**Hypertension**	**No**	*Reference*					
	**Yes**	NA	**2.19 (1.79-2.69)**	NA	**1.48 (1.25-1.75)**	NA	1.19 (0.96-1.48)

Abbreviations: LDL-C, Low-density lipoprotein cholesterol; WHO, World Health Organization; ASCVD, Atherosclerotic Cardiovascular Disease; CI, Confidence Interval; OR, Odds Ratio; BMI, Body Mass Index

* All ORs were adjusted for ethnicity as well as other variables in the column

** Criteria for good control:

*** in high risk: LDL-C < 115 mg/dL (3.0 mmol/L) and total cholesterol<190 mg/dL (5.0 mmol/L)

****in very high risk: LDL-C < 77 mg/dL (2.0 mmol/L) and total cholesterol <152 mg/dL (4.0 mmol/L)

***NA: These variables were not adjusted in the model, because they were used to calculate risk of ASCVD

Good lipid control in those who were treated with statin was associated with male sex (0.67 [0.60–0.76]), older age (1.35 [1.01–1.80]) in high-risk group and diabetes (1.23 [1.03–1.46]) in established CVD group.

## Discussion

The current study is a comprehensive estimate of dyslipidemia epidemiology across Iran. According to our findings, 73.2% of Iranian adults aged 35–70 years are affected. One in every five of them has more than 20% risk of developing cardiovascular events in the next 10 years, which makes them eligible for receiving statin treatment, regardless of their lipid values. However, 74.6% of the high risk and 58.2% of participants with CVD did not receive the recommended treatment, with 55.2% and 76.4% of them not at target lipid goals, respectively.

Comparing the prevalence of dyslipidemia in different studies is challenging, because there are diverse diagnostic criteria for lipid cutoff values and numerous definitions. As mentioned above, the prevalence of dyslipidemia in the current study was estimated according to the updated criteria of NCEP ATP III [21]. Similar criteria were used by some regional studies from Iran to assess the lipid profile; for instance, in a study among 9,847 individuals in Mashhad (northeastern Iran), 83.4% of the study population had one form of dyslipidemia and 16% were at high 10-years CVD risk (≥20%), which is comparable to our estimate [25]. In another study from Tabriz (northwestern Iran), dyslipidemia was present in 83.3% of the population [26]. Moreover, in Tehran, based on the Tehran Cohort Study, the age- and sex-standardized prevalence was estimated to be 82.7% [7]. A study in 2023, as part of WHO STEPwise approach to Surveillance (STEPS) study, reported that 81.0% of adults aged over 25 years had abnormal lipid profile [6].

At a global level, the National Health and Nutrition Examination Survey (NHANES) conducted in US from 2003–2006 reported the prevalence of dyslipidemia as 53% [27]. In China, a study in 2017 reported an estimated prevalence of 62.1% [28]. The results of a meta-analysis showed that the prevalence of dyslipidemia in the Middle East and North Africa (MENA) region has varied, ranging from 10.5% to 86.7% [29]. Congruent with our findings, the prevalence of low HDL-C, high TC and high TG are the most prominent components of lipid abnormality in this region [29]. This pattern, also known as atherogenic dyslipidemia [30], has been shown to be strongly associated with metabolic syndrome and diabetes, and may be linked to higher future CVD risk [31]. Therefore, the high prevalence of high TG and low HDL-C in our study highlights the need for addressing the determinants associated with these types of lipid abnormalities, as well as directing the focus of management from only reducing the LDL-C levels to approaches that target low HDL-C and high TG as well.

While female sex was associated with high TC and LDL-C, and low HDL-C, men had higher odds of having high TG. A study from the UK in 2015 reported men to have lower HDL-C compared to either pre- or post-menopausal women, which is in contrast with our findings [32]. A study from Turkey, a neighboring country of Iran, reported an association between male sex and high LDL-C, which is also contradictory to our findings [33]. It seems that the association between plasma lipid components and sex is highly impacted by age, menopause and other genetics and environmental risk factors, which vary regionally and study by study..

While dyslipidemia was associated negatively with aging in men, we showed that the prevalence of high TC, LDL-C and TG increased with age in women. Consistent with our findings, aging in women has been shown to be associated with higher odds of dyslipidemia [34–36]. This has been partly attributed to the link between older age and menopause in women, which has been shown to cause an increase in TC, TG, LDL-C and a decrease in HDL-C due to reduced estrogen production [37]. As a result, post-menopause women are at greater risk of developing coronary heart diseases, compared to their younger counterparts [38]. However, there seems to be other factors at play as well, since the association between age and dyslipidemia was still existent in women of our study, even after controlling for menopause variable.

We found obesity, low physical activity and low education to be significant risk factors for all components of lipid profile, which is in agreement with previous studies [39–41]. In our study, tobacco consumption was associated with lower HDL-C and higher TG. This is consistent with previous studies that suggest cigarette smoking could promote synthesis of fatty acids and suppression of lipoprotein lipase, and result in increased levels of TG and decreased levels of HDL-C, mediated through pro-inflammatory cytokines [42–45]. In our study, alcohol consumption was associated with higher TC, TG, and LDL-C. However, the literature regarding the association of alcohol and plasma lipid components frequently suggests a greater level of HDL-C as a result of reduced activity of Cholesterol Ester Transfer Protein (CETP), and neutral effects on other components [46,47]. Alcohol consumption showed a positive association with HDL-C in our study; however, it did not reach significance.

The significant cardiovascular correlates of low HDL-C and high TG in our population were tobacco consumption, low physical activity, diabetes, obesity and hypertension. Therefore, our study underscores the necessity of life-style modification and if necessary, medical treatment.

Traditionally, the primary goal of lipid disorder management has been reducing LDL-C burden to decrease the risk of developing CVD in the future, and statins have been the drug of choice [48]. Although high LDL-C was not the most prevalent lipid abnormality in our study, a significant proportion of our population remained untreated and uncontrolled. Only 24.7% of high-risk individuals and 41.8% of those with established CVD were receiving the recommended statin treatment, indicating a notable underutilization of statins. In contrast, a study by the Centers for Disease Control and Prevention (CDC) in the U.S. reported a 48% treatment rate among eligible individuals following the NCEP ATP III guidelines between 2005 and 2008 [49], Furthermore, trends in the U.S. show a steady increase in statin use over time. An analysis of National Health and Nutrition Examination Survey (NHANES) data found that the proportion of U.S. adults eligible for statin therapy who were receiving treatment increased from 14.9% in 1999–2000 to 27.8% in 2017–2018 [50]. Specifically, among adults with diabetes aged 40–75 years, statin use rose from 21.4% to 51.9% during the same period, a rate notably higher than that observed in our study [50]. However, our treatment percentages are higher than reports from China; a study from China in 2021 reported a treatment prevalence of 14% among those with established CVD and only 4.5% among high risk individuals [46]. A 2005 study by WHO MONICA (monitoring trends and determinants in cardiovascular disease) project conducted in 19 countries reported generally low prevalence of receiving hypercholesterolemia treatment around the world [51]. Even among populations with higher awareness compared to others, the level of treatment was insufficient. The study suggested the frequency of screening for high cholesterol as a key factor for increasing the chance of receiving treatment [51]. Consequently, these observations indicate a potential need for a more advanced screening program for detection of dyslipidemia in Iran..

Regarding the correlates of receiving treatment, recent studies indicate a persistent sex disparity in statin prescription, with women being less likely to receive statin therapy compared to men. For instance, the overall prescription rate for statins is significantly lower in women (67.0%) than in men (78.4%), and this disparity extends across all risk groups [52]. While we observed a similar association in the high-risk group, female sex was associated with lower treatment odds among those with established CVD. This discrepancy may be partly due to lower statin prescription rates by healthcare providers and a higher tendency for women to discontinue treatment [52]. These findings highlight the need for targeted interventions to reduce gender-based disparities in lipid management.

Regarding lipid control, only 23.6% of individuals with established CVD and 45.1% of high-risk individuals in our study achieved target lipid goals as defined by WHO guidelines. Among those receiving statin therapy, 69.6% of high-risk individuals and 41.3% of those with CVD reached adequate lipid control. Our findings align with recent global studies highlighting persistent gaps in LDL-C management, despite advancements in lipid-lowering therapies [53]. A large-scale U.S. study found that 50% of patients with ASCVD were not on any statin, while over two-thirds remained above LDL-C goals despite treatment [54]. Similarly, the GOULD registry revealed that only 17% of patients with uncontrolled LDL-C received treatment intensification over two years [55]. In Europe, the SANTORINI study reported that only 27.3% of high-risk patients were on combination therapy, with statin monotherapy remaining dominant despite guideline recommendations [56]. In a comparable pattern, the AIZANOI study in Turkey found that only 26.2% of statin-treated patients achieved guideline-recommended LDL-C targets, despite 58.4% of very high-risk and 44.4% of high-risk patients being on high-intensity statin therapy [57]. These findings underscore the urgent need to increase statin adherence, optimize treatment intensification, and promote combination therapies for high-risk individuals failing to achieve LDL-C targets.

Our study showed that women had lower odds of good lipid control, despite having higher odds of receiving treatment. Similarly, in the study by Lu et al., women had lower odds of achieving lipid control (OR: 0.58 [95% CI: 0.56–0.59] in the high-risk group and 0.56 [95% CI: 0.53–0.58] in those with CVD) [46]. Older age was also associated with better lipid control among eligible individuals, which aligns with our findings [46] Further supporting this pattern, a Swiss study analyzing primary care records of 59,092 patients found that women had lower LDL-C assessment rates than men in both primary (aOR: 0.71 [95% CI: 0.67–0.75]) and secondary prevention (aOR: 0.70 [95% CI: 0.51–0.95]). Moreover, despite receiving treatment, women had higher measured LDL-C levels compared to men (mean difference: + 0.30 mmol/L [CI: 0.25–0.35] in primary prevention and +0.28 mmol/L [CI: 0.07–0.48] in secondary prevention) [58]. These results suggest that lipid management strategies may be less effective in women, potentially due to differences in response to therapy, adherence, or physician treatment decisions. Notably, these findings contrast with a 2015 U.S. study, which suggested that male sex was a predictor of achieving LDL-C targets (OR: 1.08 [95% CI: 1.06–1.09]) [59]. The reasons behind these gender disparities in lipid control remain unclear, but they highlight the need for more targeted interventions to improve LDL-C management in women.

Alcohol consumption was associated with lower control in all eligible groups. The Lu et al. study from China also showed a lower lipid control for drinking alcohol (OR: 0.87 [95% CI: 0.83–0.92]) in those with established CVD [46]. Similar to treatment, having diabetes and hypertension were associated with better lipid control in people with established CVD. This issue has been controversial in previous studies, as some studies suggest that metabolic comorbidities such as diabetes and hypertension prevent people from achieving lipid goals [59], while some show the opposite [60,61]. The American Diabetes Association (ADA) Standards of Care underscore the significance of comprehensive lipid management in individuals with diabetes, advocating for aggressive cardiovascular risk reduction strategies to mitigate complications [62]. These guidelines may provide a potential explanation for our findings, as individuals with diabetes and hypertension may demonstrate greater adherence to treatment protocols due to heightened awareness of their cardiovascular risk. Furthermore, clinicians may be more inclined to prescribe intensive lipid-lowering therapies in this population to achieve optimal lipid control and reduce the likelihood of adverse cardiovascular events.

As mentioned above, different populations and ethnicities around the world show different patterns of dyslipidemia and plasma lipid concentrations. In the current study, we used the NCEP ATP III cut points for diagnosis of dyslipidemia and the updated WHO guideline for defining high risk individuals in need of treatment. However, these guidelines have not been validated for the Iranian population. Therefore, one of the highlights of our study is the need for developing a national guideline for management of lipid disorders; one with refined lipid values according to the population of Iran.

### Strengths and limitations

This study benefits from a large population-based sample which includes all major ethnicities spread across Iran. The cross-sectional nature of this study is the most important limitation, which makes causal inference impossible. Since the study is cross-sectional, all reported relationships should be interpreted as associations rather than causal effects. There are no specified guidelines for the management of dyslipidemia in Iran, and we had to use the most applicable guideline which is defined by the WHO. However, this guideline has some limitations. There is no definition for optimal lipid level in the low-risk group. Therefore, we cannot know the status of lipid control among our low-risk population. Also, data on awareness regarding dyslipidemia was not available in our study.

## Conclusion

In conclusion, the prevalence of dyslipidemia in our population of 35–70-year-old Iranians is extremely high. More than 20% of the population are at high risk of developing cardiovascular events in the next 10 years, which makes them eligible for receiving treatment with statin medications. Among those eligible, the majority are not achieving the recommended lipid goals required to reduce CVD risk. Certain groups are at higher risk of not receiving the treatment they need and of having poor lipid control, and this should be considered when designing and implementing national healthcare policies on the dyslipidemia management and CVD prevention.

## Supporting information

S1 Table Baseline characteristics and lipid profile distribution of the PERSIAN Cohort Study participants. (PDF)

S2 Table Weighted multivariable logistic regression model of factors associated with dyslipidemia in women, adjusted for menopause. (PDF)
