# Supplementary material for: Epidemiology and determinants of dyslipidemia in Iranian population: Results from the PERSIAN cohort
Source: PLoS One. 2026 Jul 17;21(7):e0352516. doi: 10.1371/journal.pone.0352516 (PMC13379098; doi:10.1371/journal.pone.0352516)
Supplement: S1 Table — (PDF) [file pone.0352516.s001.pdf]

Supplement to:

Epidemiology and Determinants of Dyslipidemia in Iranian Population: Results from the PERSIAN Cohort

S1 Table. Baseline characteristics and lipid profile distribution of the PERSIAN Cohort Study participants

		TC<br>Median (Q1, Q3)*	LDL-C<br>Median (Q1, Q3)	HDL-C<br>Median (Q1, Q3)	TG<br>Median (Q1, Q3)	Non-HDL-C<br>Median (Q1, Q3)
**Total**		187.5 (163, 215)	108.2 (87.6, 130.6)	48.7 (41, 57)	130 (94.5, 185)	114.4 (138, 163)
**Gender**	**Men**	183.2 (159, 210)	105.8 (85.2, 127.4)	45 (39, 53)	136 (98, 196)	137 (114, 162)
	**Women**	191 (166, 218.7)	110.2 (89.6, 133.2)	51 (44, 60)	125 (92, 175)	138.4 (115, 164)
**Age**	**35-44 years**	182 (159, 206.1)	104.24 (86, 125)	48 (41, 56)	123 (88.2, 178)	133 (111, 156.8)
	**45-54 years**	190.4 (166,218)	110.2 (89.6, 132.4)	49 (41, 57)	134 (98, 190)	141 (118, 166)
	**55-64 years**	192 (165, 221)	111.6 (88.6, 135.6)	49 (42, 58)	134 (98, 190)	142 (116, 169)
	**≥65 years**	188 (161, 218)	109.6 (85.4, 133.6)	49 (42, 58)	129 (96, 176)	138 (112, 165)
**Residence area**	**Urban**	188 (164, 215.9)	108.2 (87.7, 130.58)	49 (41, 57)	134 (98, 190)	139 (116, 164)
	**Rural**	185.67 (161, 212.2)	108.09 (87.4, 130.6)	48 (41, 57)	119 (87, 170)	136 (112, 161.5)
**Education**	**0 years**	191 (165, 220)	111.44 (89.4, 135.2)	50 (42.3, 58.3)	127.9 (93, 179)	140 (116, 167.5)
	**1-5 years**	188 (163, 215)	108.74 (88.2, 131.2)	49 (41, 57)	127 (94, 180)	138 (114, 163.4)
	**≥ 6 years**	186 (162, 212)	106.6 (86.6, 128.2)	48 (41, 56)	132 (96, 189.9)	137 (114, 162)
**Wealth Score category**	**Low**	187 (162, 214)	108.6 (87.68, 131.4)	49 (41, 57)	121.4 (88.6, 173)	136.3 (112, 163)
	**Average**	189 (165, 216)	107.6 (87.2, 130)	48 (41, 56.4)	131 (96, 187)	138 (114, 163)
	**High**	183 (158, 214)	108.4 (88.08, 130.2)	49 (41.2, 57)	137 (100, 194)	139.2 (117, 164)
**Marital status**	**Married**	187 (162.5, 214)	107.84 (87.4, 130.16)	48 (41, 57)	130 (95, 185)	138 (114, 163)
	**Unmarried**	192 (167, 221)	111.8 (90.2, 135.4)	51 (44, 60)	125 (92, 177)	140 (115, 167)
	**Never**	189 (164.7, 216)	109 (88.6, 131.6)	50 (42, 58)	128 (93, 181)	138 (115, 164)

**Tobacco consumption**	**Ever**	184 (158.5, 210)	105.86 (85, 127.8)	45 (39, 53)	135 (98, 194)	137 (113, 162.3)
**Opium consumption**	**Never**	188 (164, 215.3)	108.8 (88.2, 131.2)	49 (41.6, 57)	130 (95, 184)	138 (115, 164)
	**Ever**	180 (155, 206.6)	102.8 (82.8, 125)	45.7 (39, 53.7)	129 (93, 188)	133 (109, 159)
**Alcohol consumption**	**Never**	188 (163, 215)	108.3 (87.8, 130.8)	49 (41.1, 57)	129 (94, 183)	138 (114.1, 163)
	**Ever**	185 (161, 212)	106.8 (86, 128.4)	45 (38, 53)	141 (101, 202)	140 (116, 164)
**Physical activity**	**Low**	188 (163, 216)	108.2 (87, 131)	48 (40, 56)	138 (100, 195)	139.6 (116, 165)
	**Average**	189 (164.4, 216)	109 (88.8, 131.6)	50 (42, 58)	129 (95, 183)	138.2 (115, 164)
	**High**	185 (161, 212)	107.6 (87.2, 129.2)	48 (41, 57)	122 (89, 175)	136 (113, 161)
**BMI**	**<25**	179 (155.5, 205.5)	104.4 (84.88, 126.2)	49 (42, 58)	105 (78, 147)	128 (106, 153)
	**25-29.9**	189 (164.7, 216)	109.04 (88.4, 131.4)	48 (41, 56)	135.9 (99, 193)	140 (117, 165)
	**≥30**	193 (168, 220)	110.6 (89.4, 133.2)	49 (41, 57)	145.8 (107.1, 201)	143 (120, 168.3)
**Diabetes**	**No**	188 (164, 214)	109.2 (89.2, 131)	49 (41, 57)	125 (92, 177)	138 (115, 163)
	**Yes**	186.3 (157.4, 218)	101 (77, 127)	48 (40.3, 56)	161 (115, 226)	138 (110, 167)
**Hypertension**	**No**	186 (162.8, 213)	107.8 (88.4, 129.58)	49 (42, 57)	124 (90, 177)	136 (114, 161)
	**Yes**	190 (163.5, 217)	108.8 (86.2, 132.2)	48 (40.6, 56)	140 (101.6, 196)	140.7 (116, 167)
**Cardiovascular disease**	**No**	188 (164,215)	109.1 (89, 131.2)	49 (41, 57)	129 (94, 184)	139 (116, 164)
	**Yes**	177.25 (148, 208)	97.2 (73.4, 23.6)	48 (40, 56)	136 (100, 190)	128.4 (102, 157.8)

Abbreviations: TC, Total cholesterol; LDL-C, Low-density lipoprotein cholesterol; HDL-C, High-density lipoprotein cholesterol; TG, Triglyceride; BMI, Body Mass Index
* Q1: Quartile 1; Q3: Quartile 3
