# Supplementary material for: Epidemiology and determinants of dyslipidemia in Iranian population: Results from the PERSIAN cohort
Source: PLoS One. 2026 Jul 17;21(7):e0352516. doi: 10.1371/journal.pone.0352516 (PMC13379098; doi:10.1371/journal.pone.0352516)
Supplement: S2 Table — (PDF) [file pone.0352516.s002.pdf]

Supplement to:

Epidemiology and Determinants of Dyslipidemia in Iranian Population: Results from the PERSIAN Cohort

S2 Table. Weighted multivariable logistic regression model of factors associated with dyslipidemia in women, adjusted for menopause

		OR (95% CI)	P-value
**Age**	**35-44 years**	*Reference*	
	**45-54 years**	**1.20 (1.10-1.30)**	**<0.001**
	**55-64 years**	**1.37 (1.19-1.57)**	**<0.001**
	**≥65 years**	**1.31 (1.07-1.61)**	**0.01**
**Residence area**	**Urban**	*Reference*	
	**Rural**	091 (0.80-1.04)	0.165
**Education**	**0 years**	*Reference*	
	**1-5 years**	1.01 (0.95-1.09)	0.664
	**≥ 6 years**	0.90 (0.79-1.03)	0.118
**Wealth Score index category**	**Low**	*Reference*	
	**Average**	0.98 (0.87-1.12)	0.809
	**High**	0.96 (0.83-1.12)	0.635
**Marital status**	**Unmarried**	*Reference*	
	**Married**	1.02 (0.95-1.10)	0.584
**Tobacco consumption**	**Never**	*Reference*	
	**Ever**	**1.19 (1.09-1.30)**	**<0.001**
**Opium consumption**	**Never**	*Reference*	
	**Ever**	0.82 (0.64-1.04)	0.1

**Alcohol consumption**	**Never**	*Reference*	
	**Ever**	0.88 (0.60-1.28)	0.492
**Physical activity**	**Low**	*Reference*	
	**Average**	**0.85 (0.80-0.91)**	**<0.001**
	**High**	**0.72 (0.67-0.77)**	**<0.001**
**BMI**	**<25**	*Reference*	
	**25-29.9**	**1.74 (1.63-1.85)**	**<0.001**
	**≥30**	**2.28 (2.13-2.445)**	**<0.001**
**Diabetes**	**No**	*Reference*	
	**Yes**	**2.20 (2.00-2.42)**	**<0.001**
**Hypertension**	**No**	*Reference*	
	**Yes**	**1.37 (1.26-1.49)**	**<0.001**
**Cardiovascular disease**	**No**	*Reference*	
	**Yes**	**1.24 (1.08-1.41)**	**0.003**
**Menopause**	**No**	*Reference*	
	**Yes**	**1.39 (1.28-1.51)**	**<0.001**

Abbreviations: OR, Odds Ratio; CI, Confidence Interval; BMI, Body Mass Index

* ORs were adjusted for all variables in the column as well as ethnicity
