# Supplementary material for: Epidemiology and determinants of dyslipidemia in Iranian population: Results from the PERSIAN cohort
Source: PLoS One. 2026 Jul 17;21(7):e0352516. doi: 10.1371/journal.pone.0352516 (PMC13379098; doi:10.1371/journal.pone.0352516)
Supplement: Attachment — Submitted filename: RTR_2.docx [file pone.0352516.s003.docx]

Dear Joven Serdoncillo

Thank you indeed for considering our manuscript entitled “Epidemiology and Determinants of Dyslipidemia in Iranian Population: Results from the PERSIAN Cohort”.

Per the instructions we received, we have made the following changes in our submission:

Before we can proceed, we have noted that the IRB document you have supplied does not match the approval number stated in your manuscript (IR.TUMS.DDRI.REC.1396.1). Please could you address this and provide the correct information/documentation for the study.

**Response:** Thanks for your attention. The approval number was wrong in the manuscript. Instead of (IR.TUMS.DDRI.REC.1396.1), it was corrected into (IR.TUMS.DDRI.REC.1392.001)
