# Supplementary material for: Epidemiology and determinants of dyslipidemia in Iranian population: Results from the PERSIAN cohort
Source: PLoS One. 2026 Jul 17;21(7):e0352516. doi: 10.1371/journal.pone.0352516 (PMC13379098; doi:10.1371/journal.pone.0352516)
Supplement: Attachment — Submitted filename: RTR_May_2026.docx [file pone.0352516.s005.docx]

Dear Dr. Shayo

We really appreciate that you are reconsidering our manuscript entitled “Epidemiology and Determinants of Dyslipidemia in Iranian Population: Results from the PERSIAN Cohort” for publication in PLOS One.

**Response to journal requirements**

1) None of the reviewers recommended a specific published work to cite. We checked the reference list and we ensure it is complete and correct.
2) Unfortunately, we cannot make our data publicly available for two reasons: restrictions imposed by the Iran’s National Higher Security Council, which strictly prohibits sharing any data with organizations abroad, and the fact that the data are owned by other medical universities as third parties.

**Response to the fourth comment of Reviewer #1.**

Reviewer #1: 4. You stated that “Systolic blood pressure (SBP) was defined as the average of systolic blood pressures of right and left arms measured for the second time. Diastolic blood pressure (DBP) was defined as the average of diastolic blood pressures of right and left arms measured for the second time.“ Please justify using this method or include an appropriate reference to support it.

Note: I do not see this justification in the main text. Please include it with an appropriate reference.

**Response:** Thank you very much indeed for this invaluable comment. The following paragraph was added to the methods section, corresponding to lines 218 to 224 in the clean manuscript:

“Blood pressure was measured twice in both arms, and systolic blood pressure (SBP) and diastolic blood pressure (DBP) were defined as the mean of second‑cycle measurements from the right and left arms, to reduce random measurement variability. Persistent inter‑arm differences are known to predict increased cardiovascular risk, and current recommendations advise using the higher arm for clinical decision-making; however, in this epidemiologic analysis the averaged value was used as a continuous exposure measure. Our choice to average is a conscious methodological decision within an epidemiologic context rather than a contradiction of clinical management guidelines. This approach is consistent with the protocol of the PERSIAN study.”
