# Supplementary material for: Epidemiology and determinants of dyslipidemia in Iranian population: Results from the PERSIAN cohort
Source: PLoS One. 2026 Jul 17;21(7):e0352516. doi: 10.1371/journal.pone.0352516 (PMC13379098; doi:10.1371/journal.pone.0352516)
Supplement: Peer review history

# Author comment: Author response to Decision Letter 0

**DOI:** 10.1371/journal.pone.0352516.r001

21 Sep 2025

Attachment Submitted filename: RTR_2.docx

# Peer review: Decision Letter 0

**DOI:** 10.1371/journal.pone.0352516.r002

**Authors:** Hossein Ali Adineh

6 Nov 2025

PONE-D-25-46624

Epidemiology and Determinants of Dyslipidemia in Iranian Population: Results from the PERSIAN Cohort

PLOS ONE

Dear Dr. Malekzadeh,

Thank you for submitting your manuscript to PLOS ONE. After careful consideration, we feel that it has merit but does not fully meet PLOS ONE’s publication criteria as it currently stands. Therefore, we invite you to submit a revised version of the manuscript that addresses the points raised during the review process.

Please submit your revised manuscript by Dec 21 2025 11:59PM. If you will need more time than this to complete your revisions, please reply to this message or contact the journal office at plosone@plos.org. When you're ready to submit your revision, log on to https://www.editorialmanager.com/pone/ and select the 'Submissions Needing Revision' folder to locate your manuscript file.

Please include the following items when submitting your revised manuscript:

- A rebuttal letter that responds to each point raised by the academic editor and reviewer(s). You should upload this letter as a separate file labeled 'Response to Reviewers'.
- A marked-up copy of your manuscript that highlights changes made to the original version. You should upload this as a separate file labeled 'Revised Manuscript with Track Changes'.
- An unmarked version of your revised paper without tracked changes. You should upload this as a separate file labeled 'Manuscript'.

If you would like to make changes to your financial disclosure, please include your updated statement in your cover letter. Guidelines for resubmitting your figure files are available below the reviewer comments at the end of this letter.

If applicable, we recommend that you deposit your laboratory protocols in protocols.io to enhance the reproducibility of your results. Protocols.io assigns your protocol its own identifier (DOI) so that it can be cited independently in the future. For instructions see: https://journals.plos.org/plosone/s/submission-guidelines#loc-laboratory-protocols. Additionally, PLOS ONE offers an option for publishing peer-reviewed Lab Protocol articles, which describe protocols hosted on protocols.io. Read more information on sharing protocols at https://plos.org/protocols?utm_medium=editorial-email&utm_source=authorletters&utm_campaign=protocols.

We look forward to receiving your revised manuscript.

Kind regards,

Hossein Ali Adineh, Ph.D

Academic Editor

PLOS ONE

Journal requirements:

When submitting your revision, we need you to address these additional requirements.

1. Please ensure that your manuscript meets PLOS ONE's style requirements, including those for file naming. The PLOS ONE style templates can be found at

https://journals.plos.org/plosone/s/file?id=wjVg/PLOSOne_formatting_sample_main_body.pdf and

https://journals.plos.org/plosone/s/file?id=ba62/PLOSOne_formatting_sample_title_authors_affiliations.pdf

2. Your ethics statement should only appear in the Methods section of your manuscript. If your ethics statement is written in any section besides the Methods, please move it to the Methods section and delete it from any other section. Please ensure that your ethics statement is included in your manuscript, as the ethics statement entered into the online submission form will not be published alongside your manuscript.

3. If the reviewer comments include a recommendation to cite specific previously published works, please review and evaluate these publications to determine whether they are relevant and should be cited. There is no requirement to cite these works unless the editor has indicated otherwise.

Additional Editor Comments (if provided):

[Note: HTML markup is below. Please do not edit.]

Reviewers' comments:

Reviewer's Responses to Questions

**Comments to the Author**

1. Is the manuscript technically sound, and do the data support the conclusions?

The manuscript must describe a technically sound piece of scientific research with data that supports the conclusions. Experiments must have been conducted rigorously, with appropriate controls, replication, and sample sizes. The conclusions must be drawn appropriately based on the data presented.

Reviewer #1: Partly

Reviewer #2: Yes

**********

2. Has the statistical analysis been performed appropriately and rigorously?

Reviewer #1: Yes

Reviewer #2: Yes

**********

3. Have the authors made all data underlying the findings in their manuscript fully available?

The PLOS Data policy requires authors to make all data underlying the findings described in their manuscript fully available without restriction, with rare exception (please refer to the Data Availability Statement in the manuscript PDF file). The data should be provided as part of the manuscript or its supporting information, or deposited to a public repository. For example, in addition to summary statistics, the data points behind means, medians and variance measures should be available. If there are restrictions on publicly sharing data—e.g. participant privacy or use of data from a third party—those must be specified.

Reviewer #1: No

Reviewer #2: No

**********

4. Is the manuscript presented in an intelligible fashion and written in standard English?

PLOS ONE does not copyedit accepted manuscripts, so the language in submitted articles must be clear, correct, and unambiguous. Any typographical or grammatical errors should be corrected at revision, so please note any specific errors here.

Reviewer #1: No

Reviewer #2: Yes

**********

5. Review Comments to the Author

Please use the space provided to explain your answers to the questions above. You may also include additional comments for the author, including concerns about dual publication, research ethics, or publication ethics. (Please upload your review as an attachment if it exceeds 20,000 characters)

Reviewer #1: The manuscript is generally well-prepared; however, several points require attention and clarification.

1. Please ensure that all references follow the Vancouver citation style, using square brackets [ ] throughout the manuscript. For detailed formatting requirements, refer to the PLOS ONE journal submission guidelines: https://journals.plos.org/plosone/s/submission-guidelines?utm_source=chatgpt.com.

2. Please explain the sampling technique used to select clusters and the method applied to choose individuals within each cluster for participation in the study. In addition, provide information on the participation refusal rate, including its percentage and the main reasons for refusal.

3. In the main text of your manuscript, you mentioned that a questionnaire was conducted; however, its purpose, structure, and results were not clearly described. In addition, there is no mention of the questionnaire in the abstract. Please provide further clarification about the questionnaire in both the abstract and the main text.

4. You stated that “Systolic blood pressure (SBP) was defined as the average of systolic blood pressures of right and left arms measured for the second time. Diastolic blood pressure (DBP) was defined as the average of diastolic blood pressures of right and left arms measured for the second time.“. Please justify using this method or include an appropriate reference to support it.

5. Hypertension (HTN) was defined as SBP ≥ 140 mmHg, and/or DBP ≥ 90 mmHg. Please provide the reference that supports this classification.

6. Each figure should include a complete caption, consisting of a title followed by a legend that explains the figure or provides additional details. Please ensure that a legend is added after the title in all figure captions.

7. Please minimize repetition of certain terms throughout the manuscript:

- Reduce the frequent use of the term “dyslipidemia.” For instance, in the sentence “Findings of this study highlight the necessity of developing a national guideline for management of dyslipidemia.” you may replace the repeated term with “the disease” to improve readability.

- Similarly, try to limit the repeated use of “CVD” to make the text more fluent and professional.

8. Create a new section titled “Supporting Information” and list all supplementary materials there to make it easier for readers to review them.

9. It seems that the Acknowledgment section is missing. Kindly add it to the manuscript if applicable.

Reviewer #2: Peer Review Report

General Overview

The manuscript presents an analysis based on a large, nationally representative dataset from the Persian Cohort Study. The objective was to examine patterns and predictors of dyslipidemia across different Iranian ethnic groups, and to assess treatment coverage and lipid control among individuals at high cardiovascular risk, according to the updated World Health Organization (WHO) guidelines for CVD prevention.

The study relies on robust data and appropriate analytical methods, including multivariable regression with clearly reported ORs and CIs. .

The major strengths of the present work are:

Large, nationally representative sample: The study is based on a substantial dataset from the Persian Cohort Study, allowing for robust population-level inferences and ethnic subgroup comparisons.

Relevant research question: Investigating dyslipidemia patterns and predictors across different ethnicities in Iran addresses an important public health issue and contributes valuable region-specific evidence to global CVD prevention research.

Use of appropriate analytical framework: The authors employ complex survey design and multivariable logistic regression models, which are suitable for the study objectives and large-scale population data.

Clear reporting of key associations:ORs and confidence intervals CIs are systematically presented, and the main findings—particularly differences by age, sex, and ethnicity—are statistically and epidemiologically meaningful.

Potential for significant impact: The study provides actionable insights into treatment gaps and lipid control among high-risk groups, which could inform national cardiovascular prevention strategies.

Major Comments

Organization and Structure: The Methods and Results sections are overly dense and lack clear subheadings. All methodological steps (sampling, weighting, descriptive analysis, regression modeling) are presented in a single block, making it difficult to follow. The Results section should also be divided into logical subsections (e.g., Prevalence by Age and Sex, Determinants of Dyslipidemia, Statin Use and Lipid Control). The writing style is heavy and repetitive, with long sentences that reduce clarity.

Incomplete Methodological Details: Key analytical information is missing, including model specifications, adjustment variables, and definitions of “good control.” The explanation of sampling weights is inadequate: there is no indication of adjustment for nonresponse, post-stratification, or calibration. The implementation of clustering and stratification in Stata is not described. Covariates included in multivariable models should be listed explicitly.

Model Diagnostics and Statistical Rigor: No mention is made of interaction testing, collinearity diagnostics, or model validation (e.g., Hosmer–Lemeshow test, VIF). No adjustments for multiple testing are reported (e.g., Bonferroni or FDR correction). The use of an outdated software version (Stata 12) limits reproducibility and modern survey analysis capabilities.

Data Quality and Missing Data: 2,008 participants (1.2%) were excluded due to missing lipid values, but no bias assessment was performed. There is no mention of outlier detection, data cleaning procedures, or sensitivity analyses to assess robustness.

Interpretation of Results: Several associations are reported with an implied causal tone, although the study design is cross-sectional. Ethnic differences are presented ambiguously without adjustment details or contextual interpretation (socio-cultural or behavioral factors).

Outcome Definition: The term “good control” of lipid levels is not defined according to established cut-off thresholds for TC or LDL-C. This must be clarified.

Redundancy: Predictors such as education, BMI, and physical activity are repeatedly described in multiple models without synthesis or summarization.

Minor Comments

Formatting and Typographical Issues : Numerous typographical artifacts and nonstandard decimal separators should be corrected. Statistical notations (p-values, confidence intervals) should be standardized. Language and Style: Simplify complex or repetitive phrasing. For examplke, Use concise, active constructions ( “We described baseline characteristics” instead of “We used descriptive analysis to demonstrate baseline characteristics”).

**********

6. PLOS authors have the option to publish the peer review history of their article (what does this mean?). If published, this will include your full peer review and any attached files.

If you choose “no”, your identity will remain anonymous but your review may still be made public.

**Do you want your identity to be public for this peer review?**  For information about this choice, including consent withdrawal, please see our Privacy Policy.

Reviewer #1: No

Reviewer #2: No

**********

[NOTE: If reviewer comments were submitted as an attachment file, they will be attached to this email and accessible via the submission site. Please log into your account, locate the manuscript record, and check for the action link "View Attachments". If this link does not appear, there are no attachment files.]

To ensure your figures meet our technical requirements, please review our figure guidelines: https://journals.plos.org/plosone/s/figures

You may also use PLOS’s free figure tool, NAAS, to help you prepare publication quality figures: https://journals.plos.org/plosone/s/figures#loc-tools-for-figure-preparation.

NAAS will assess whether your figures meet our technical requirements by comparing each figure against our figure specifications.

# Author comment: Author response to Decision Letter 1

**DOI:** 10.1371/journal.pone.0352516.r003

31 Jan 2026

Dear Dr. Adineh,

We really appreciate that you are reconsidering our manuscript entitled “Epidemiology and Determinants of Dyslipidemia in Iranian Population: Results from the PERSIAN Cohort” for publication in PLOS One. We would like to thank the editors and reviewers for careful and thorough review of this manuscript, which helped us to improve its quality. The manuscript has been revised for better readability according to the suggestions of the reviewers and editors. We hope it reaches your standards for publication in the journal.

We disclose that AI-assisted language-editing tools were used solely for grammatical revision and stylistic improvements. No AI system was used to generate data, perform analyses, or contribute to the scientific content. All intellectual responsibility for the works remains with the authors.

This cover letter includes a point-by-point response to the comments. The changes are also highlighted in the main manuscript.

Sincerely yours,

Reza Malekzadeh

Journal requirements:

When submitting your revision, we need you to address these additional requirements.

1. Please ensure that your manuscript meets PLOS ONE's style requirements, including those for file naming. The PLOS ONE style templates can be found at https://journals.plos.org/plosone/s/file?id=wjVg/PLOSOne_formatting_sample_main_body.pdf

and

https://journals.plos.org/plosone/s/file?id=ba62/PLOSOne_formatting_sample_title_authors_affiliations.pdf

Response: Thanks indeed for mentioning the requirements. We revised the manuscript to meet PLOS ONE’s style requirements.

2. Your ethics statement should only appear in the Methods section of your manuscript. If your ethics statement is written in any section besides the Methods, please move it to the Methods section and delete it from any other section. Please ensure that your ethics statement is included in your manuscript, as the ethics statement entered into the online submission form will not be published alongside your manuscript.

Response: The ethics statement is mentioned only in the methods section.

3. If the reviewer comments include a recommendation to cite specific previously published works, please review and evaluate these publications to determine whether they are relevant and should be cited. There is no requirement to cite these works unless the editor has indicated otherwise.

Response: Neither of the reviewers recommended to cite any specific published works.

Review Comments to the Author

Please use the space provided to explain your answers to the questions above. You may also include additional comments for the author, including concerns about dual publication, research ethics, or publication ethics. (Please upload your review as an attachment if it exceeds 20,000 characters)

Reviewer #1: The manuscript is generally well-prepared; however, several points require attention and clarification.

1. Please ensure that all references follow the Vancouver citation style, using square brackets [ ] throughout the manuscript. For detailed formatting requirements, refer to the PLOS ONE journal submission guidelines: https://journals.plos.org/plosone/s/submission-guidelines?utm_source=chatgpt.com.

Response: Thanks indeed for this comment. The references were revised according to the style of PLOS One.

2. Please explain the sampling technique used to select clusters and the method applied to choose individuals within each cluster for participation in the study. In addition, provide information on the participation refusal rate, including its percentage and the main reasons for refusal.

Response: Thanks for this important comment. The following section was added to the manuscript:

“Cohort sites were selected from both urban and rural areas based on the following criteria: representation of Iran’s major ethnic groups, inclusion of diverse climates to capture varied environmental and occupational exposures, low migration rates to minimize follow-up loss, and previously reported local disease patterns. Men and women aged 35–70 years residing in the PERSIAN Cohort regions were invited to participate. In smaller cities, all eligible individuals were approached, while in larger cities or multiethnic areas, specific districts were selected to reflect socioeconomic and ethnic diversity. Participants attended the cohort’s recruitment centers and all eligible individuals were enrolled. Eligibility required Iranian descent and residence in the designated area for at least nine months annually. Individuals with physical or psychological conditions preventing completion of enrollment were excluded.”

And the following paragraph:

“To invite the eligible participants, trained fieldworkers went door to door describing the study objectives and encouraging qualified individuals for participation and referral to the cohort’s recruitment center. Therefore, it was not possible to record non-response.”

However, to address the absence of refusal data: “We used sampling weights defined as the inverse probability of being selected in the survey based on data of the national census in 2016, to obtain weighted estimates that reflect the underlying target population.”

3. In the main text of your manuscript, you mentioned that a questionnaire was conducted; however, its purpose, structure, and results were not clearly

described. In addition, there is no mention of the questionnaire in the abstract. Please provide further clarification about the questionnaire in both the abstract and the main text.

Response: Thanks indeed for this comment. The following section was added to the abstract:

“Participants’ characteristics were recorded using validated questionnaires including biological samples, physical measurements, and self-reported information.”

The following section was added to the methods of the manuscript:

“Questionnaires were administered face-to-face by trained interviewers using the standard PERSIAN Cohort instrument, which includes 482 items across general, medical, and nutritional domains. The questionnaire collects detailed sociodemographic, medical, lifestyle, and dietary information, including a validated food-frequency questionnaire. Data were entered online into a centralized database and checked for completeness by field supervisors, and several questionnaire components (e.g., socioeconomic status, physical activity, circadian rhythm, and the FFQ) have been validated within the cohort.”

4. You stated that “Systolic blood pressure (SBP) was defined as the average of systolic blood pressures of right and left arms measured for the second time. Diastolic blood pressure (DBP) was defined as the average of diastolic blood pressures of right and left arms measured for the second time.“ Please justify using this method or include an appropriate reference to support it.

Response: Thanks very much indeed for this invaluable comment.

Blood pressure was measured twice in both arms, and systolic and diastolic blood pressures for analysis were defined as the mean of second cycle measurements from the right and left arms, to reduce random measurement variability. Persistent inter arm differences are known to predict increased cardiovascular risk, and current recommendations advise using the higher arm for clinical decision-making; however, in this epidemiologic analysis the averaged value was used as a continuous exposure measure. Our choice to average is a conscious methodological decision within an epidemiologic context rather than a contradiction of clinical management guidelines.

5. Hypertension (HTN) was defined as SBP ≥ 140 mmHg, and/or DBP ≥ 90 mmHg. Please provide the reference that supports this classification.

Response: Thanks for this invaluable comment. Although the ACC/AHA defined hypertension as SBP>=130 mmHg and DBP>=80 mmHg, the European Society of Cardiology (ESC), and European and International Society of Hypertension (ESH/ISH) blood pressure guidelines have maintained SBP>=140 mmHg and DBP>=90 mmHg to define hypertension due to its impact on definition of hypertension prevalence and treatment targets. Still, the definition of BP>=140/90 is considered as stage II hypertension in the guideline of ACC/AHA as well. The references are as follows and they were added to the list of references:

1) Jones DW, Ferdinand KC, Taler SJ, Johnson HM, Shimbo D, Abdalla M, et al. 2025 AHA/ACC/AANP/AAPA/ABC/ACCP/ACPM/AGS/AMA/ASPC/NMA/PCNA/SGIM Guideline for the Prevention, Detection, Evaluation and Management of High Blood Pressure in Adults: A Report of the American College of Cardiology/American Heart Association Joint Committee on Clinical Practice Guidelines. Circulation. 2025;152(11):e114-e218. Epub 20250814. doi: 10.1161/cir.0000000000001356. PubMed PMID: 40811497.

2) van den Born BH, Bejan-Angoulvant T, Pengo MF, Mahfoud F, Sudano I, Hanssen H, et al. Targets for Treatment and Optimal Strategies for Non-Pharmacological and Pharmacological Management of Hypertension. European journal of preventive cardiology. 2025. Epub 20250923. doi: 10.1093/eurjpc/zwaf610. PubMed PMID: 40985288.

6. Each figure should include a complete caption, consisting of a title followed by a legend that explains the figure or provides additional details. Please ensure that a legend is added after the title in all figure captions.

Response: Thanks indeed for this comment. The caption was added to the figures as follows:

Figure-1: Weighted prevalence of different components of dyslipidemia in the PERSIAN Cohort Study population. Weighted prevalence (%) of each dyslipidemia component in the study population. Estimates are based on standardized biochemical measurements; abbreviations include TC, TG, LDL-C, HDL-C, and non-HDL-C.

Figure-2: Weighted prevalence of different components of dyslipidemia in different age and sex groups. Weighted prevalence (%) of dyslipidemia components stratified by age group and sex. Component definitions and weighting methods follow the study protocol.

Figure 3: The percentage of lipid control among different ASCVD risk groups. ASCVD: Atherosclerotic Cardiovascular Disease. Percentage of participants achieving lipid-control targets within each ASCVD risk group. Definitions of lipid-control thresholds and ASCVD categories follow the study protocol.

7. Please minimize repetition of certain terms throughout the manuscript:

- Reduce the frequent use of the term “dyslipidemia.” For instance, in the sentence “Findings of this study highlight the necessity of developing a national guideline for management of dyslipidemia.” you may replace the repeated term with “the disease” to improve readability.

- Similarly, try to limit the repeated use of “CVD” to make the text more fluent and professional.

Response: Thanks indeed for this comment. The entire manuscript was revised accordingly. However, we doubt that dyslipidemia is actually considered a “disease”

8. Create a new section titled “Supporting Information” and list all supplementary materials there to make it easier for readers to review them.

Response: The list of supplementary materials was added to the end of the manuscript.

9. It seems that the Acknowledgment section is missing. Kindly add it to the manuscript if applicable.

Response: Thanks for this comment. The acknowledgment was added to the manuscript.

Reviewer #2: Peer Review Report

Major Comments

Organization and Structure: The Methods and Results sections are overly dense and lack clear subheadings. All methodological steps (sampling, weighting, descriptive analysis, regression modeling) are presented in a single block, making it difficult to follow. The Results section should also be divided into logical subsections (e.g., Prevalence by Age and Sex, Determinants of Dyslipidemia, Statin Use and Lipid Control). The writing style is heavy and repetitive, with long sentences that reduce clarity.

Response: Thank you for this helpful comment. The Methods section is reorganized into the following subsections: Study setting, Data collection, Definition of variables, and Statistical analyses, with each analytic step (sampling, weighting, descriptive analysis, and regression modeling) clearly separated and briefly introduced.

The Results section has also been restructured into logically labeled subsections, including Prevalence of dyslipidemia, Correlates of dyslipidemia, and Statin use and lipid control, to improve readability and flow.

In addition, long and repetitive sentences throughout the Methods, Results, and Discussion have been shortened and simplified, and redundant phrasing has been removed to enhance clarity.

Incomplete Methodological Details: Key analytical information is missing, including model specifications, adjustment variables, and definitions of “good control.” The explanation of sampling weights is inadequate: there is no indication of adjustment for nonresponse, post-stratification, or calibration. The implementation of clustering and stratification in Stata is not described. Covariates included in multivariable models should be listed explicitly.

Response: Thanks for this comment. Additional details have been added to the Statistical analyses subsection to fully describe the model specifications and covariate adjustment. Specifically, for each multivariable logistic regression model, we have explicitly listed all covariates (sociodemographic, lifestyle, and clinical factors) and indicate which models exclude certain variables because they are components of the WHO CVD risk score.

The definition of good control of lipid levels has been clarified in the Methods section, with explicit TC and LDL C cut off thresholds for each risk group, according to the WHO guideline used, and these same thresholds are referenced in the Results.

The description of sampling weights has been expanded to explain that weights were derived as the inverse probability of selection based on the 2016 national census to obtain weighted estimates that reflect the underlying target population, and we now state explicitly that we did not perform additional nonresponse or post stratification adjustments beyond those incorporated in the PERSIAN Cohort design.

We now describe how the survey design was implemented in Stata, including the use of the svyset command with primary sampling units and strata, and the application of survey weights for all prevalence estimates and regression analyses.

Finally, for multivariable models (correlates of dyslipidemia, correlates of statin use, and correlates of lipid control), we added a concise sentence summarizing which covariates were included, and this is cross referenced in the table footnotes.

This paragraph was added to the methods section:

All models included age category, sex, residence area, education, wealth index category, marital status, physical activity, BMI category, alcohol consumption, tobacco consumption, opium use, diabetes, hypertension, and ethnicity. Tobacco use, diabetes, and hypertension were not entered in high risk models because they contribute to the estimated WHO CVD risk score.

Model Diagnostics and Statistical Rigor: No mention is made of interaction testing, collinearity diagnostics, or model validation (e.g., Hosmer–Lemeshow test, VIF). No adjustments for multiple testing are reported (e.g., Bonferroni or FDR correction). The use of an outdated software version (Stata 12) limits reproducibility and modern survey analysis capabilities.

Response: We have now included a paragraph in the Statistical analyses subsection describing the model diagnostics performed. Specifically, we report that potential multi-collinearity was assessed using variance inflation factors, which did not exceed a threshold of 5 indicating absence of collinearity.

We also state that overall model fit for the logistic regressions was evaluated using the Hosmer–Lemeshow goodness of fit test (applied on unweighted models as recommended) and that models demonstrated adequate calibration.

Regarding interaction assessment, we have clarified that we explored sex by age interactions for overall dyslipidemia and key components, and that the significant interaction motivated the sex stratified analyses.

Concerning multiple testing, we acknowledge in the Methods and Discussion th

Attachment Submitted filename: RTR_2_auresp_1.docx

# Peer review: Decision Letter 1

**DOI:** 10.1371/journal.pone.0352516.r004

**Authors:** Hossein Ali Adineh, Festo Shayo

5 May 2026

PONE-D-25-46624R1

Epidemiology and Determinants of Dyslipidemia in Iranian Population: Results from the PERSIAN Cohort

PLOS One

Dear Dr. Malekzadeh,

Thank you for submitting your manuscript to PLOS ONE. After careful consideration, we feel that it has merit but does not fully meet PLOS ONE’s publication criteria as it currently stands. Therefore, we invite you to submit a revised version of the manuscript that addresses the points raised during the review process.

Please submit your revised manuscript by Jun 19 2026 11:59PM. If you will need more time than this to complete your revisions, please reply to this message or contact the journal office at plosone@plos.org. When you're ready to submit your revision, log on to https://www.editorialmanager.com/pone/ and select the 'Submissions Needing Revision' folder to locate your manuscript file.

Please include the following items when submitting your revised manuscript:

- A letter that responds to each point raised by the academic editor and reviewer(s). You should upload this letter as a separate file labeled 'Response to Reviewers'.
- A marked-up copy of your manuscript that highlights changes made to the original version. You should upload this as a separate file labeled 'Revised Manuscript with Track Changes'.
- An unmarked version of your revised paper without tracked changes. You should upload this as a separate file labeled 'Manuscript'.

If you would like to make changes to your financial disclosure, please include your updated statement in your cover letter. Guidelines for resubmitting your figure files are available below the reviewer comments at the end of this letter.

If applicable, we recommend that you deposit your laboratory protocols in protocols.io to enhance the reproducibility of your results. Protocols.io assigns your protocol its own identifier (DOI) so that it can be cited independently in the future. For instructions see: https://journals.plos.org/plosone/s/submission-guidelines#loc-laboratory-protocols. Additionally, PLOS ONE offers an option for publishing peer-reviewed Lab Protocol articles, which describe protocols hosted on protocols.io. Read more information on sharing protocols at https://plos.org/protocols?utm_medium=editorial-email&utm_source=authorletters&utm_campaign=protocols.

As the corresponding author, your ORCID iD is verified in the submission system and will appear in the published article. PLOS supports the use of ORCID, and we encourage all coauthors to register for an ORCID iD and use it as well. Please encourage your coauthors to verify their ORCID iD within the submission system before final acceptance, as unverified ORCID iDs will not appear in the published article. *Only*  the individual author can complete the verification step; PLOS staff *cannot*  verify ORCID iDs on behalf of authors.

We look forward to receiving your revised manuscript.

Kind regards,

Festo Casmir Shayo, M.D, M.Med, PhD.

Academic Editor

PLOS One

Journal Requirements:

If the reviewer comments include a recommendation to cite specific previously published works, please review and evaluate these publications to determine whether they are relevant and should be cited. There is no requirement to cite these works unless the editor has indicated otherwise.

Please review your reference list to ensure that it is complete and correct. If you have cited papers that have been retracted, please include the rationale for doing so in the manuscript text, or remove these references and replace them with relevant current references. Any changes to the reference list should be mentioned in the rebuttal letter that accompanies your revised manuscript. If you need to cite a retracted article, indicate the article’s retracted status in the References list and also include a citation and full reference for the retraction notice.

[Note: HTML markup is below. Please do not edit.]

Reviewers' comments:

Reviewer's Responses to Questions

**Comments to the Author**

1. If the authors have adequately addressed your comments raised in a previous round of review and you feel that this manuscript is now acceptable for publication, you may indicate that here to bypass the “Comments to the Author” section, enter your conflict of interest statement in the “Confidential to Editor” section, and submit your "Accept" recommendation.

Reviewer #1: (No Response)

Reviewer #2: All comments have been addressed

**********

2. Is the manuscript technically sound, and do the data support the conclusions?

The manuscript must describe a technically sound piece of scientific research with data that supports the conclusions. Experiments must have been conducted rigorously, with appropriate controls, replication, and sample sizes. The conclusions must be drawn appropriately based on the data presented.

Reviewer #1: (No Response)

Reviewer #2: Yes

**********

3. Has the statistical analysis been performed appropriately and rigorously?

Reviewer #1: (No Response)

Reviewer #2: Yes

**********

4. Have the authors made all data underlying the findings in their manuscript fully available?

The PLOS Data policy requires authors to make all data underlying the findings described in their manuscript fully available without restriction, with rare exception (please refer to the Data Availability Statement in the manuscript PDF file). The data should be provided as part of the manuscript or its supporting information, or deposited to a public repository. For example, in addition to summary statistics, the data points behind means, medians and variance measures should be available. If there are restrictions on publicly sharing data—e.g. participant privacy or use of data from a third party—those must be specified.

Reviewer #1: (No Response)

Reviewer #2: No

**********

5. Is the manuscript presented in an intelligible fashion and written in standard English?

PLOS ONE does not copyedit accepted manuscripts, so the language in submitted articles must be clear, correct, and unambiguous. Any typographical or grammatical errors should be corrected at revision, so please note any specific errors here.

Reviewer #1: (No Response)

Reviewer #2: Yes

**********

6. Review Comments to the Author

Please use the space provided to explain your answers to the questions above. You may also include additional comments for the author, including concerns about dual publication, research ethics, or publication ethics. (Please upload your review as an attachment if it exceeds 20,000 characters)

Reviewer #1: 4. You stated that “Systolic blood pressure (SBP) was defined as the average of systolic blood pressures of right and left arms measured for the second time. Diastolic blood pressure (DBP) was defined as the average of diastolic blood pressures of right and left arms measured for the second time.“. Please justify using this method or include an appropriate reference to support it.

Note: I do not see this justification in the main text. Please include it with an appropriate reference.

Reviewer #2: Dear author,

Many thanks for your revision. All comments have been answered.

I don't have any new remarks.

**********

7. PLOS authors have the option to publish the peer review history of their article (what does this mean?). If published, this will include your full peer review and any attached files.

If you choose “no”, your identity will remain anonymous but your review may still be made public.

**Do you want your identity to be public for this peer review?**  For information about this choice, including consent withdrawal, please see our Privacy Policy.

Reviewer #1: No

Reviewer #2: No

**********

[NOTE: If reviewer comments were submitted as an attachment file, they will be attached to this email and accessible via the submission site. Please log into your account, locate the manuscript record, and check for the action link "View Attachments". If this link does not appear, there are no attachment files.]

To ensure your figures meet our technical requirements, please review our figure guidelines: https://journals.plos.org/plosone/s/figures

You may also use PLOS’s free figure tool, NAAS, to help you prepare publication quality figures: https://journals.plos.org/plosone/s/figures#loc-tools-for-figure-preparation.

NAAS will assess whether your figures meet our technical requirements by comparing each figure against our figure specifications.

# Author comment: Author response to Decision Letter 2

**DOI:** 10.1371/journal.pone.0352516.r005

1 Jun 2026

Dear Dr. Shayo

We really appreciate that you are reconsidering our manuscript entitled “Epidemiology and Determinants of Dyslipidemia in Iranian Population: Results from the PERSIAN Cohort” for publication in PLOS One.

Response to journal requirements

1) None of the reviewers recommended a specific published work to cite. We checked the reference list and we ensure it is complete and correct.

2) Unfortunately, we cannot make our data publicly available for two reasons: restrictions imposed by the Iran’s National Higher Security Council, which strictly prohibits sharing any data with organizations abroad, and the fact that the data are owned by other medical universities as third parties.

Response to the fourth comment of Reviewer #1.

Reviewer #1: 4. You stated that “Systolic blood pressure (SBP) was defined as the average of systolic blood pressures of right and left arms measured for the second time. Diastolic blood pressure (DBP) was defined as the average of diastolic blood pressures of right and left arms measured for the second time.“ Please justify using this method or include an appropriate reference to support it.

Note: I do not see this justification in the main text. Please include it with an appropriate reference.

Response: Thank you very much indeed for this invaluable comment. The following paragraph was added to the methods section, corresponding to lines 218 to 224 in the clean manuscript:

“Blood pressure was measured twice in both arms, and systolic blood pressure (SBP) and diastolic blood pressure (DBP) were defined as the mean of second cycle measurements from the right and left arms, to reduce random measurement variability. Persistent inter arm differences are known to predict increased cardiovascular risk, and current recommendations advise using the higher arm for clinical decision-making; however, in this epidemiologic analysis the averaged value was used as a continuous exposure measure. Our choice to average is a conscious methodological decision within an epidemiologic context rather than a contradiction of clinical management guidelines. This approach is consistent with the protocol of the PERSIAN study.”

Attachment Submitted filename: RTR_May_2026.docx

# Peer review: Decision Letter 2

**DOI:** 10.1371/journal.pone.0352516.r006

**Authors:** Hossein Ali Adineh, Festo Shayo, Festo Shayo

11 Jun 2026

Epidemiology and Determinants of Dyslipidemia in Iranian Population: Results from the PERSIAN Cohort

PONE-D-25-46624R2

Dear Dr. Malekzadeh,

We’re pleased to inform you that your manuscript has been judged scientifically suitable for publication and will be formally accepted for publication once it meets all outstanding technical requirements.

Within one week, you’ll receive an e-mail detailing the required amendments. When these have been addressed, you’ll receive a formal acceptance letter and your manuscript will be scheduled for publication.

An invoice will be generated when your article is formally accepted. Please note, if your institution has a publishing partnership with PLOS and your article meets the relevant criteria, all or part of your publication costs will be covered. Please make sure your user information is up-to-date by logging into Editorial Manager at Editorial Manager® and clicking the ‘Update My Information' link at the top of the page. For questions related to billing, please contact billing support.

If your institution or institutions have a press office, please notify them about your upcoming paper to help maximize its impact. If they’ll be preparing press materials, please inform our press team as soon as possible -- no later than 48 hours after receiving the formal acceptance. Your manuscript will remain under strict press embargo until 2 pm Eastern Time on the date of publication. For more information, please contact onepress@plos.org.

Kind regards,

Festo Casmir Shayo, M.D, M.Med, PhD.

Academic Editor

PLOS One

Additional Editor Comments (optional):

Reviewers' comments:

Reviewer's Responses to Questions

**Comments to the Author**

1. If the authors have adequately addressed your comments raised in a previous round of review and you feel that this manuscript is now acceptable for publication, you may indicate that here to bypass the “Comments to the Author” section, enter your conflict of interest statement in the “Confidential to Editor” section, and submit your "Accept" recommendation.

Reviewer #1: All comments have been addressed

**********

2. Is the manuscript technically sound, and do the data support the conclusions?

The manuscript must describe a technically sound piece of scientific research with data that supports the conclusions. Experiments must have been conducted rigorously, with appropriate controls, replication, and sample sizes. The conclusions must be drawn appropriately based on the data presented.

Reviewer #1: (No Response)

**********

3. Has the statistical analysis been performed appropriately and rigorously?

Reviewer #1: (No Response)

**********

4. Have the authors made all data underlying the findings in their manuscript fully available?

The PLOS Data policy requires authors to make all data underlying the findings described in their manuscript fully available without restriction, with rare exception (please refer to the Data Availability Statement in the manuscript PDF file). The data should be provided as part of the manuscript or its supporting information, or deposited to a public repository. For example, in addition to summary statistics, the data points behind means, medians and variance measures should be available. If there are restrictions on publicly sharing data—e.g. participant privacy or use of data from a third party—those must be specified.

Reviewer #1: (No Response)

**********

5. Is the manuscript presented in an intelligible fashion and written in standard English?

PLOS ONE does not copyedit accepted manuscripts, so the language in submitted articles must be clear, correct, and unambiguous. Any typographical or grammatical errors should be corrected at revision, so please note any specific errors here.

Reviewer #1: (No Response)

**********

6. Review Comments to the Author

Please use the space provided to explain your answers to the questions above. You may also include additional comments for the author, including concerns about dual publication, research ethics, or publication ethics. (Please upload your review as an attachment if it exceeds 20,000 characters)

Reviewer #1: (No Response)

**********

7. PLOS authors have the option to publish the peer review history of their article (what does this mean?). If published, this will include your full peer review and any attached files.

If you choose “no”, your identity will remain anonymous but your review may still be made public.

**Do you want your identity to be public for this peer review?**  For information about this choice, including consent withdrawal, please see our Privacy Policy.

Reviewer #1: No

**********

# Editor report: Acceptance letter

**DOI:** 10.1371/journal.pone.0352516.r007

**Authors:** Hossein Ali Adineh, Festo Shayo, Festo Shayo

PONE-D-25-46624R2

PLOS One

Dear Dr. Malekzadeh,

I'm pleased to inform you that your manuscript has been deemed suitable for publication in PLOS One. Congratulations! Your manuscript is now being handed over to our production team.

At this stage, our production department will prepare your paper for publication. This includes ensuring the following:

* All references, tables, and figures are properly cited

* All relevant supporting information is included in the manuscript submission,

* There are no issues that prevent the paper from being properly typeset

You will receive further instructions from the production team, including instructions on how to review your proof when it is ready. Please keep in mind that we are working through a large volume of accepted articles, so please give us a few days to review your paper and let you know the next and final steps.

Lastly, if your institution or institutions have a press office, please let them know about your upcoming paper now to help maximize its impact. If they'll be preparing press materials, please inform our press team within the next 48 hours. Your manuscript will remain under strict press embargo until 2 pm Eastern Time on the date of publication. For more information, please contact onepress@plos.org.

You will receive an invoice from PLOS for your publication fee after your manuscript has reached the completed accept phase. If you receive an email requesting payment before acceptance or for any other service, this may be a phishing scheme. Learn how to identify phishing emails and protect your accounts at https://explore.plos.org/phishing.

If we can help with anything else, please email us at customercare@plos.org.

Thank you for submitting your work to PLOS ONE and supporting open access.

Kind regards,

PLOS ONE Editorial Office Staff

on behalf of

Dr. Festo Casmir Shayo

Academic Editor

PLOS One
